# Supplementary material for: Enhancer–promoter interactions and transcription are largely maintained upon acute loss of CTCF, cohesin, WAPL or YY1
Source: Nat Genet. 2022 Dec 5;54(12):1919–32. doi: 10.1038/s41588-022-01223-8 (PMC9729117; doi:10.1038/s41588-022-01223-8)
Supplement: Supplementary file 1 — Supplementary Notes and Methods. [file 41588_2022_1223_MOESM1_ESM.pdf]

nature genetics

Article

https://doi.org/10.1038/s41588-022-01223-8

Enhancer–promoter interactions and transcription are largely maintained upon acute loss of CTCF, cohesin, WAPL or YY1

In the format provided by the authors and unedited

Supplementary Methods

Stable cell line construction

Cell lines stably expressing 3xFLAG-HaloTag-YY1 and YY1-HaloTag-3xFLAG were generated using PiggyBac transposition and drug selection. Full details in Supplementary Methods. The coding sequences of YY1 and 3xFLAG-HaloTag or HaloTag-3xFLAG were cloned by Gibson Assembly into a PiggyBac vector co-expressing a puromycin resistance gene. For each construct, YY1 and HaloTag were connected by a short peptide linker sequence (GDGAGLING). 1.3 μg of PiggyBac vector was co-transfected with 0.5 μg of SuperPiggyBac transposase vector into JM8.N4 cells by nucleofection using the Lonza Mouse Embryonic Stem Cell Nucleofector Kit reagents and the Amaxa Nucleofector II device. After 24 hr, antibiotic selection was begun with 500 ng/mL of puromycin. Once cells reached ~80% confluence, 5000 cells were seeded onto a 15 cm plate and allowed to grow into colonies. After 4-6 days, single clones were isolated and seeded onto a 96-well plate. Cells were allowed to expand for 4-5 days, and successfully integrated PiggyBac constructs were validated by PCR genotyping. We selected clone PBYN2 for 3xFLAG-HaloTag-YY1 and clone PBYC3 for YY1-HaloTag-3xFLAG. We carried out the same approach to generate cell lines stably expressing 3xFLAG-HaloTag-SOX2 and 3xFLAG-HaloTag-KLF4 in RAD21-AID cells, and selected clones SHT22 (SOX2) and KHT26 (KLF4) for the downstream analysis.

For all single-molecule experiments, cells were grown overnight on Matrigel-coated 25-mm circular no 1.5H cover glasses (High-Precision 0117650). Prior to all experiments, the cover glasses were plasma-cleaned and then stored in isopropanol until use. Cells were labeled with 50 nM PA-JFX646 (fast SPT) or 25 pM JFX549 (slow SPT) HaloTag ligand for 30 min and washed twice with PBS. After the final washes, cells were replenished with phenol-free medium prior to imaging.

Generation of CRISPR/Cas9-mediated knock-in cell lines

Endogenously tagged mESC lines were generated by CRISPR/Cas9-mediated genome editing as previously described[1] with modifications. mESCs were seeded to 6-well plates and each well was transfected with Lipofectamine 3000 (ThermoFisher #L3000015) or nucleofection (Lonza #VPH-1001) using 1 μg of a construct encoding Cas9, Venus yellow fluorescent protein, and a sgRNA and 2 μg of a homology repair plasmid with the DNA insert of interest flanked by left and right homology arms (~500 bp each). For each insertion we designed 2-4 individual sgRNAs with the CRISPOR tool[2] to be transfected separately into mESCs and pooled before sorting Venus-positive cells by FACS. Sorted cells were plated at a density of ~5,000-10,000 cells per P15 plate and media replaced every other day. Single colonies were transferred to 96-well plates to be expanded and genotyped by PCR after crude DNA extraction with the DirectPCR Lysis Reagent (Viagen Biotech #302-C). Clones with a correct insert were further verified by Sanger sequencing and Western blot. The parental cell line of CTCF, Rad21 and WAPL auxin-inducible degron (AID) mESCs was clone C59 from Hansen et al.[3] further modified to visualize DNA looping (Clone C36 osTir1: see Gabriele et al.[4] for full details on the generation of C36).

To endogenously tag 1) CTCF, RAD21, or WAPL with mAID[5] in the C36 osTir mESC line; 2) YY1 with miniIAA7[6] in the parental JM8.N4 with osTIR1 expression; 3) YY1 with HaloTag in the parental JM8.N4; and 4) CTCF and RAD21 with mAID in the YY1-HaloTag cell lines, we designed homology repair plasmids encoding 1) 3xFLAG-BFP-mAID-CTCF, RAD21-BFP-mAID-V5, HA-BFP-mAID-WAPL; 2) miniIAA7-mScarletI-YY1; 3) 3xFLAG-HaloTag-YY1; 4) 3xFLAG-mNeonGreen-mAID-CTCF and RAD21-mNeonGreen-mAID-V5 (plasmids, sgRNAs and primer sequences in **Supplementary Table 1**). Homozygous clones with the correct genotype were further verified by Sanger sequencing and Western blot after IAA-mediated depletion. Among the several clones generated for each AID line, we picked clone C58 for 3xFLAG-BFP-mAID-CTCF; clone F1 for RAD21-BFP-mAID-V5; clone C40 for HA-BFP-mAID-WAPL; clone YD39 for miniIAA7-mScarletI-YY1; clone YN11 for 3xFLAG-HaloTag-YY1; clone CD1 for 3xFLAG-mNeonGreen-mAID-CTCF and clone RD35 for RAD21-mNeonGreen-mAID-V5.

Western Blotting

For Western blot analysis, cells were seeded to 6-well plates, washed with ice-cold PBS and scraped in 300 μl of low-salt lysis buffer (0.1 M NaCl, 25 mM HEPES, 1 mM MgCl2, 0.2 mM EDTA, 0.5% NP-40 and protease inhibitors), with 125 U/mL of benzonase (Novagen, EMD Millipore), rocked at 4 °C for 1 hr and then NaCl was added to a 0.2-M final concentration. Lysates were then rocked at 4 °C for 30 min and centrifuged at maximum speed at 4 °C. Supernatants were quantified by Bradford. Alternatively, cells were lysed directly on plates with 300 μl of high-salt lysis buffer (0.5 M NaCl, 25 mM HEPES, 1 mM MgCl2, 0.2 mM EDTA, 0.5% NP-40 and protease inhibitors) and transferred to low-stick tubes with 100 μl of 4x SDS-loading buffer. Proteins were loaded onto a 9% Bis-Tris SDS-PAGE gel, transferred onto a nitrocellulose membrane (Amershan Protran 0.45 um NC, GE Healthcare) for 1 hr at 100V, blocked in TBS-Tween with 10% milk for at least 1 hr at room temperature and blotted overnight at 4 °C with primary antibodies in TBS-T with 5% milk (antibodies listed in **Supplementary Table 1**). HRP-conjugated secondary antibodies were diluted 1:5000 in TBS-T with 5% milk and incubated with the membrane at room temperature for an hour before performing the chemiluminescence reaction (Western Lightning Plus-ECL, Enhanced Chemiluminescence Substrate, Perkin Elmer NEL105001EA). Signal was captured with either X-ray films (CL-XPosure™ Film, ThermoScientific 34091) or with a Bio-Rad ChemiDoc imaging system.

Chromatin immunoprecipitation (ChIP) and ChIP-seq

ChIP was performed as described with the few modifications described here[7]. Cells were treated with either ethanol or 500 mM of ethanol-dissolved auxin for 3 hrs and cross-linked for 7 min at room temperature with 1% formaldehyde-containing medium; cross-linking was stopped by PBS-glycine (0.125 M final). Cells were washed twice with ice-cold PBS, scraped, centrifuged for 10 min and pellets were flash-frozen. Cell pellets were thawed and resuspended in cell lysis buffer (5 mM PIPES, pH 8.0, 85 mM KCl, and 0.5% NP-40, 1 ml/15 cm plate) with protease inhibitors and incubated for 10 min on ice. Lysates were centrifuged for 10 min at 1250 x g and nuclear pellets

were resuspended in 6 volumes of sonication buffer (50 mM Tris-HCl, pH 8.1, 10 mM EDTA, 0.1% SDS) with protease inhibitors, incubated on ice for 10 min, and sonicated to obtain DNA fragments below 2000 bp in length (Covaris S220 sonicator, 20% Duty factor, 200 cycles/burst, 150 peak incident power, 10-20 cycles 50 sec on and 30 sec off). Sonicated lysates were cleared by centrifugation and chromatin (400 μg per antibody) was diluted in RIPA buffer (10 mM Tris-HCl, pH 8.0, 1 mM EDTA, 0.5 mM EGTA, 1% Triton X-100, 0.1% SDS, 0.1% Na-deoxycholate, 140 mM NaCl) with protease inhibitors to a final concentration of 0.8 μg/μl, precleared with Protein G sepharose (GE Healthcare) for 2 hrs at 4°C and immunoprecipitated overnight with 4 μg of specific antibodies (antibodies listed in **Supplementary Table 1**). About 4% of the precleared chromatin was saved as input. Immunoprecipitated DNA was purified with the Qiagen QIAquick PCR Purification Kit, eluted in 36 μl of 0.1X TE (1 mM Tris-HCl pH 8.0, 0.01 mM EDTA) and analyzed by qPCR together with 2% of the input chromatin prior to ChIP-seq library preparation (SYBR® Select Master Mix for CFX, ThermoFisher; ChIP-qPCR primer sequences in **Supplementary Table 1**).

ChIP-seq libraries were prepared using the NEBNext® Ultra™ II DNA Library Prep Kit for Illumina® (NEB E7645) according to manufacturer instructions with a few modifications. 20 ng of ChIP input DNA (as measured by Nanodrop) and 25 μl of the immunoprecipitated DNA were used as a starting material and the recommended reagent volumes were cut in half. Some of the YY1 ChIP-seq libraries in the YY1 degron line (Clone YD39) were prepared adding 50 pg of MNase digested yeast DNA as a spike-in control to both inputs and ChIP samples. The NEBNext Adaptor for Illumina was diluted 1:10 in Tris/NaCl, pH 8.0 (10 mM Tris-HCl pH 8.0, 10 mM NaCl) and the ligation step extended to 30 min. After ligation, a single purification step with 0.9X volumes of Agencourt AMPure XP PCR purification beads (Beckman Coulter A63880) was performed, eluting DNA in 22 μl of 10 mM Tris-HCl pH 8.0. 20 μl of the eluted DNA was used for the library enrichment step, performed with the KAPA HotStart PCR kit (Roche Diagnostics KK2502) in 50 μl of total reaction volume (10 μl 5X KAPA buffer, 1.5 μl 10 mM dNTPs, 0.5 μl 10 μM NEB Universal PCR primer, 0.5 μl 10 μM NEB index primer, 1 μl KAPA polymerase, 16.5 μl nuclease-free water and 20 μl sample). Samples were enriched with 9 PCR cycles (98 °C, 45 sec; [98 °C, 15 sec; 60 °C, 10 sec] x 9; 72 °C, 1 min; 4 °C, hold), purified with 0.9 volumes of AMPure XP PCR purification beads and eluted with 33 μl of 10 mM Tris-HCl pH 8.0.

ChIP-seq raw reads from ethanol (UT)- or auxin (IAA)-treated ΔCTCF, ΔRAD21, ΔWAPL and ΔYY1 mESCs (96 libraries total, 2 biological replicates per condition plus 12 spiked-in libraries for YY1 ChIP in ΔYY1 clone YD39) were quality-checked with FastQC 0.10.1 and aligned onto the mouse genome (mm10 assembly) using Bowtie 2.3.0[8], allowing for two mismatches (-n 2) and no multiple alignments (-m 1). For spiked-in libraries, ChIP-seq raw reads were also aligned onto the yeast genome (sacCer3 assembly) using the same Bowtie parameters. Biological replicates were pooled and peaks were called with MACS2 2.1.0.20140616 (`--nomodel --extsize`

`250`)[9] using input DNA as a control. To create heatmaps we used deepTools 2.4.1[10]. We first ran bamCoverage function (`--binSize 50 --normalizeTo1x 2150570000 --extendReads 300 --ignoreDuplicates -of bigwig`) and normalized read numbers to 1x sequencing depth, obtaining read coverage per 50-bp bins across the whole genome (bigWig files). For spiked-in libraries, the number of deduplicated, unique reads aligning to the yeast genome in each sample was used to compute the bamCoverage scale factor to perform spike-in normalization. We then used the bigWig files to compute read numbers across 6 kb centered on either CTCF, RAD21, or YY1 peak summits as called by MACS2 in UT cells (`computeMatrix reference-point --referencePoint=TSS --upstream 3000 --downstream 3000 --missingDataAsZero --sortRegions=no`). We sorted the output matrices by decreasing UT enrichment, calculated as the total number of reads within a MACS2 called ChIP-seq peak. Finally, heatmaps were created with the plotHeatmap function (`--averageTypeSummaryPlot=mean --colorMap='Blues' --sortRegions=no`). To identify the differential peaks between UT- and IAA-treated cells, we used MAnorm2[11], which employs a hierarchical strategy for normalization of ChIP-seq data and assesses within-group variability of ChIP-seq signals based on an empirical Bayes framework. We note that the total ChIP-seq peak numbers in MAnorm2 are combined from UT- and IAA-treated cells and may differ from the number of MACS2 calling.

Micro-C assay for mammalian cells

We briefly summarize the Micro-C experiment here. The detailed protocol and technical discussion are available in our previous study[12]. Mouse embryonic stem cells (JM8.N4) and the derivative genome-edited lines were cultured in the recommended conditions[13]. When cells grew to ~70% confluency, we resuspended them in 0.05% of trypsin, inactivated with cell culture media, and resuspended in 1% formaldehyde crosslinking media (without FBS). Cells were crosslinked for 10 min at room temperature (RT) while nutating. We then added 1 M Tris-HCl pH 7.5 (final concentration 375 mM) and incubated for 5 min at RT to quench the crosslinking reaction. Cells were spun down and washed twice with cold PBS. Cell pellets were crosslinked again with the freshly prepared 3 mM DSG crosslinking solution (in base media without FBS) for 45 min at RT while nutating. The crosslinking reaction was quenched and washed following the same steps as above. We routinely split cells into 1 million cells per vial after fixation and perform MNase titration and Micro-C with 1 million cells. Crosslinked cell pellets can be snap frozen in liquid nitrogen and stored at -80 °C for months or used immediately for the next step. We note that 1) cells directly crosslinked on the dish typically yield a similar result; 2) using freshly made formaldehyde and DSG solution is critical to obtain high-quality Micro-C data; and 3) to avoid loss of cells, low-retention tubes and tips are strongly recommended.

Crosslinked cell pellets were resuspended in MB1 (50 mM NaCl, 10 mM Tris-HCl pH 7.5, 5 mM $MgCl_2$, 1 mM $CaCl_2$, 0.2% NP-40) at a concentration of $1x10^6$ cells/100 µL and were incubated for 20 min on ice. Cells were spun down and washed once with MB1. We then added the appropriate amount of Micrococcal nuclease (MNase) and incubated the tube for 10 min at 37 °C

while shaking in a thermomixer at ~850 rpm. The optimal digestion condition results in ~90% of mono-nucleosome and ~10% of di-nucleosome. The MNase reaction was inactivated by adding 4 mM EGTA and incubated for 10 min at 65 °C. Digested chromatin was spun down and washed twice with 1 mL of cold MB2 (50 mM NaCl, 10 mM Tris-HCl pH 7.5, 10 mM $MgCl_2$). We note that and MNase titration that yields 90% monomer/10% dimers substantially reduces contamination with un-digested (un-ligated) dimers in Micro-C data.

MNased-fragmented chromatin was then subjected to the three-step end-repair protocol to generate ligatable ends filled with biotin-dNTPs. First, the pellet was resuspended in the end-repair buffer (50 mM NaCl, 10 mM Tris-HCl pH 7.5, 10 mM $MgCl_2$, 100 µg/mL BSA, 2 mM ATP, 5 mM DTT) and the 5'-ends were phosphorylated with 25 units of T4 Polynucleotide Kinase (NEB #M0201) for 15 min at 37 °C while shaking in a thermomixer at 1000 rpm for an interval of 15 sec every 3 min. Second, to convert the mixed types of nucleosomal ends to cohesive ends, we added 25 units of DNA Polymerase I, Large (Klenow) Fragment (NEB #M0210) directly to the reaction and incubated the tube for an additional 15 min at 37 °C while shaking in a thermomixer at 1000 rpm for an interval of 15 sec every 3 min. Third, to repair the nucleosomal DNA ends to the blunt and ligatable ends, we supplemented 66 µM of dNTPs (dTTP, dGTP (NEB #N0446), biotin-dATP (Jena Bioscience #NU-835-BIO14), biotin-dCTP (Jena Bioscience #NU-809-BIOX), and 1X T4 DNA ligase reaction buffer (NEB #B0202) directly into the reaction and incubated for 45 min at 25 °C while shaking in a thermomixer at 1000 rpm for an interval of 15 sec every 3 min. The end-repair reaction was then inactivated with 30 mM EDTA for 20 min at 65°C without shaking. Next, chromatin was pelleted by centrifugation for 5 min at ~10,000xg at 4 °C and washed once with cold MB3 (50 mM Tris-HCl pH 7.5, 10 mM $MgCl_2$).

End-repaired nucleosomes were then subjected to proximity ligation with ~5000 cohesive end units (CEU) of T4 DNA ligase (NEB #M0202) in 1X T4 DNA ligase reaction buffer (NEB #B0202) for at least 2 hrs at room temperature with slow rotation on an orbital shaker. Biotin-dNTPs at the unligated DNA termini were removed by ~500 units of Exonuclease III (NEB #M0206) in 1X NEBuffer 1 (NEB #B7001) for 15 min at 37°C while shaking in a thermomixer at 1000 rpm for an interval of 15 sec every 3 min. Samples were then reverse crosslinked with 1X proteinase K solution (500 ug/uL Proteinase K (ThermoFisher #AM2542), 1% SDS, 0.1 µg/µL RNaseA) at 65°C overnight. DNA was extracted by the standard phenol:chloroform:isoamyl alcohol (25:24:1) and ethanol precipitation procedure. DNA was then purified again with the ZymoClean DNA Clean & Concentrator-5 Kit (Zymo #D4013). Purified DNA was separated on a 3% NuSieve GTG agarose gel (Lonza #50081). The gel band corresponding to the size of dinucleosomal DNA (~250 to 350 bp) was cut and purified with the Zymoclean Gel DNA Recovery Kit (Zymo #D4008). We note that size selection for DNA larger than 200 bp greatly reduces the ratio of unligated monomers in Micro-C data.

Micro-C sequencing libraries were generated by using the NEBNext Ultra II DNA Library Prep Kit for Illumina (NEB #E7645) with the following modifications. We first repaired the purified DNA again using the End-it DNA End-Repair Kit (Lucigen #ER0720) following the manufacturer's suggested conditions. The mix was incubated for 45 min at 25 °C and then

inactivated the enzyme reaction for 10 min at 70 °C. This step is optional, but we find it increases the library yield. Biotinylated DNA was captured by Dynabeads MyOne Streptavidin C1 beads (ThermoFisher #65001) in 1X BW buffer (5 mM Tris-HCl pH 7.5, 0.5 mM EDTA, 1 M NaCl) on a nutator for 20 min at room temperature. Beads were washed twice with 1X TBW buffer (0.1% Tween20, 5 mM Tris-HCl pH 7.5, 0.5 mM EDTA, 1 M NaCl) for 5 min at 55 °C while shaking in a thermomixer at 1200 rpm, rinsed once with Tris buffer (10 mM Tris-HCl pH 7.5), and then resuspended in Tris buffer.

We then performed 'on-bead' end-repair/A tailing and adapter ligation following the NEB protocol. After adapter ligation, beads were washed once with 1X TBW and rinsed once with Tris buffer. The Micro-C library was generated by using the KAPA HiFi HotStart ReadyMix (Roche #KK2601) or the Q5 High-Fidelity 2X Master Mix (NEB #E7645) with the manufacturer's suggested conditions. We recommend using a minimal PCR cycle to reduce PCR duplicates, typically 8 to 12 cycles, which can generate high-quality Micro-C data. Prior to sequencing, purifying the library twice with 0.85X AMPure XP beads (Beckman #A63880) can eliminate primer dimers and adapters. We used Illumina 100 bp paired-end sequencing (PE100) to obtain ~400M reads for each replicate in this study.

Micro-C data processing and analyses

Valid Micro-C contact read pairs were obtained from the HiC-Pro analysis pipeline (v2.11.3)[14], and the detailed description and code can be found at https://github.com/nservant/HiC-Pro. In brief, paired fastq files were mapped to the mouse mm10 genome independently using Bowtie 2.3.0 with 'very-sensitive-local' mode[8]. Aligned reads were paired by the read names. Pairs with multiple hits, low MAPQ, singleton, dangling end, self-circle, and PCR duplicates were discarded using Samtools (v.1.9). Paired reads with distances shorter than 100 bp (e.g., unligated mono-nucleosome) were also removed. Output files containing all valid pairs were used in downstream analyses. We recommend running a pilot sequencing run (~10M reads) and checking the following quality control statistics before moving forward to a high-coverage sequencing: (1) bowtie mapping rate; (2) reads pairing percentage; (3) ratio of sequencing artifacts; (4) ratio of cis/trans contacts; (5) unligated monomer percentage. If any of the above statistics is not optimal, one might consider checking mapping and filtering parameters or further optimizing the Micro-C experiment. The summary of Micro-C interactions in this manuscript is available in **Supplemental Table 2**.

We evaluated the reproducibility and data quality for the Micro-C replicates using two published methods independently (https://github.com/kundajelab/3DChromatin_ReplicateQC)[15]. In brief, QuASAR-QC (v1.5.7) calculates the correlation of values in two distance-based transformed matrices. GenomeDISCO (v1.0.0) measures the difference in two graph diffusion-smoothed contact maps. We computed the matrix similarity scores between the biological replicates or between the untreated and IAA-treated cells for the 10-kb, 25-kb, 50-kb, and 250-kb Micro-C data.

The detailed descriptions can be found in Sauria et al. for QuASAR-QC[16] and Ursu et al. for GenomeDISCO[17].

To identify chromatin domains (TADs) along the diagonal, we used insulation score analysis from the Cooltools package (v0.8.10) (https://github.com/mirnylab/cooltools) or arrowhead transformation analysis from the JUICER package (v1.22.01) (https://github.com/aidenlab/juicer)[18]. The detailed methods were described in Crane et al. for the insulation score analysis[19] or Rao et al. for the arrowhead transformation analysis[20]. Briefly, we analyzed the insulation profile by using a 1-Mb sliding window that scans across Micro-C contact matrices at 20-kb resolution and assigns an insulation intensity to its corresponding bin. The insulation scores were obtained and normalized as the log2 ratio of the individual score to the mean of the genome-wide averaged insulation score. Chromatin boundaries can be identified by finding the local minima along with the normalized insulation score. Boundaries overlapping with low mappability regions were removed from the downstream analysis. The arrowhead analysis defines $A_{i,i+d} = (M^*_{i,i-d} – M^*_{i,i+d})/(M^*_{i,i-d} + M^*_{i,i+d})$, where M* is the normalized contact matrix. $A_{i,i+d}$ can be thought of as the measurement of the directionality preference of locus *i*, restricted to contacts at a linear distance of *d*. $A_{i,i+d}$ will be strongly positive/negative if either *i,i-d* or *i,i+d* is inside the domain and the other is not, but $A_{i,i+d}$ will be close to zero if both loci are inside or outside the domain. By assigning this query across the genome, the edges of a domain will be sharpened and TADs can be detected. For aggregate domain analysis (ADA), each domain was rescaled to a pseudo-size by $N_{i,j}=((C_i-D_{start})/(D_{end}-D_{start}), (C_j-D_{start})/(D_{end}-D_{start}))$, where $C_{i,j}$ is a pair of contact loci within domain D that is flanked by D_{start} and D_{end}, and $N_{i,j}$ is a pair of the rescaled coordinates. The rescaled domains can be aggregated at the center of the plot with ICE or distance normalization. Coolpup (v0.9.5) (https://github.com/open2c/coolpuppy)[21] has implemented a handy function to perform ADA with the COOL file. The lists of TAD called by the insulation score analysis or Arrowhead are available in **Supplemental Table 3 or 4**.

To identify loops/dots, we tested two novel algorithms, Mustache (v1.0.1) (https://github.com/ay-lab/mustache)[22] and Chromosight (v0.9.8) (https://github.com/koszullab/chromosight)[23], for the high-resolution Micro-C data. We found that both approaches outperform the HICCUPS algorithm in the JUICER package[18] and the 'Call-dots' function in the Cooltools package in sensitivity and specificity to discover focal contact enrichment. In Mustache analysis, we called loops with balanced contact matrices at resolutions of 400 bp, 600 bp, 800 bp, 1 kb, 2 kb, 4 kb, 10 kb, and 20 kb using the calling options `--pThreshold 0.1 --sparsityThreshold 0.88 --octaves 2`. We then combined all loops at different resolutions. If an interaction was detected as a loop at different resolutions, we retained the precise coordinates in finer resolutions and discarded the coarser resolution. In Chromosight analysis, we used the 'detect' function to call loops with balanced contact matrices at resolutions of 400 bp, 600 bp, 800 bp, 1 kb, 2 kb, 4 kb, 10 kb, and 20 kb using calling options listed in **Supplemental Table 5**. We then combined all loops at different resolutions by the same approach as described above. We applied the lists of loop

anchor to many downstream analyses by using Bedtools (v2.30.0)[24], R (v4.0), Python (v3.8), or MATLAB (v2021a), including (1) comparison of loop anchors between Micro-C and Hi-C or between different chromatin states; (2) distribution of loop strength or length; (3) cross-correlation with ChIP-seq, RNA-seq, and mNET-seq data; (4) ratio of boundary crossing, etc. For analysis of paired genomic loci (e.g., paired ChIP-seq peaks, genetic features, etc.) within a distance ranging from 2 kb to 2 Mb, we used Chromosight's 'quantify' function to measure the probability of loop pattern for all intersections quantitatively. The loops were filtered by the following parameters: loop score >0.35 for 10-kb resolution, loop score >0.3 for 4-kb resolution, loop score >0.2 for 2-kb resolution, and the q-value lower than 10^{-5} for all resolutions (**Supplemental Table 5**). For aggregate peak analysis (APA) to assess genome-wide loop intensity, loops were centered and piled up on a 20-kb x 20-kb matrix with 400-bp resolution balanced data or 50-kb x 50-kb matrix with 1-kb resolution balanced data. Contacts close to the diagonal were excluded and normalized by a random shift matrix to avoid distance decay effects. The ratio of loop enrichment was calculated by dividing normalized center contacts in a searching window by the normalized corner submatrices. We used the same approach and normalization method to analyze the genome-wide target-centered loop intensity. Instead of aggregating at the intersection of loop anchors, the matrix is centered at the paired ChIP-seq peaks or genomic features. Coolpup (v0.9.5) (https://github.com/open2c/coolpuppy)[21] has implemented the APA function for the COOL file. The lists of loops called by Mustache or Chromosight are available in **Supplemental Table 6 or 7**. The lists of loop quantification for cohesin, E-P, and P-P loops are available in **Supplemental Table 8 – 10.**

Nascent RNA-seq experiment and analysis

We used the nascent RNA-seq (mNET-seq) protocol described in Nojima et al[25] with the following minor changes. In brief, the chromatin fraction was purified from $1x10^7$ mES cells by the following procedure: 1) Wash cells with cold PBS twice; 2) Resuspend cells with 4 mL cold HLB+N (10mM Tris-HCl (pH 7.5), 10 mM NaCl, 2.5 mM $MgCl_2$ and 0.5 % NP-40) and incubate for 5 min on ice; 3) Add 1 ml cold HLB+NS (10 mM Tris-HCl (pH 7.5), 10 mM NaCl, 2.5 mM $MgCl_2$, 0.5 % NP-40 and 10 % Sucrose) under the layer of cell lysate; 4) Spin down cells and collect the nuclear pellet; 5) Add 120 µL cold NUN1 (20 mM Tris-HCl (pH 7.9), 75 mM NaCl, 0.5 mM EDTA and 50 % Glycerol) and transfer sample to a new tube; 6) Add 1.2 mL cold NUN2 (20 mM HEPES-KOH (pH 7.6), 300 mM NaCl, 0.2 mM EDTA, 7.5 mM $MgCl_2$, 1 % NP-40 and 1 M Urea) and incubate for 15 min on ice while vortexing every 3 min; 7) Spin down the chromatin pellet and wash with cold PBS once. Next, chromatin and RNA were digested in 100 µL MNase digestion solution (1x micrococcal nuclease (MNase) buffer and 40 units/µl MNase (NEB, #M0247)) for 5 min at 37 °C while shaking at 1,400 rpm in a thermomixer. 25 mM EGTA was then added to inactivate the reaction. Digested/solubilized chromatin was then collected by centrifugation at 16,000xg for 5 min at 4 °C. The chromatin bound-Pol II complex was purified by the following steps: 1) Dilute 100 µL of sample with 400 µL cold NET-2 (50 mM Tris-HCl (pH 7.4), 150 mM NaCl, 0.05 % NP-40 and 1% Empigen BB (Sigma, cat no. 30326)); 2) Add 100 µL Pol II antibody-

conjugated beads (10 µg Pol II 8WG16 antibody conjugated with Dynabeads™ Protein G for Immunoprecipitation (ThermoFisher, #1004D)) and incubate on a tube rotator for 1 hour in the 4 °C room; 3) Wash beads with 1 mL cold NET-2 for a total of 6 washes. RNA was then phosphorylated with T4 polynucleotide kinase (T4 PNK) with the following steps: 1) Wash beads with 50 µL cold 1X PNKT buffer (1X T4 PNK buffer and 0.1 % Tween); 2) Resuspend beads with 50 µL PNK reaction mixture (1X T4 PNKT, 1 unit/µL PNK and 1 mM ATP) for 5 min at 37 °C while shaking at 1,200 rpm in a thermomixer; 3) Wash beads with 1 mL cold NET-2. RNA was isolated by the standard TRIzol RNA extraction protocol with isopropanol RNA precipitation. Purified RNA was then dissolved in 10 µL of Urea dye (7 M Urea, 0.05 % Xylene cyanol, 0.05 % Bromophenol blue) and resolved on a 6% TBE-Urea gel at 200 V for 5 min. To size select 30-160 nt RNAs, we cut the gel between the Bromophenol blue and the Xylene cyanol dye markers. A 0.5-mL tube was punctured with 3-4 small holes by 26G needle and inserted in a 1.5-mL tube. Gel fragments were placed in the 0.5-mL tube and broken down by centrifugation at 16,000xg for 1 min. RNA was eluted by RNA elution buffer (1 M NaOAc and 1 mM EDTA) for 1 hr at 25°C in a Thermomixer shaking at 900 rpm in a thermomixer. Eluted RNA was purified with SpinX column (Coster, #8160) with a glass filter (Whatman, #1823-010). The eluted RNA was purified again with ethanol precipitation.

Single-particle imaging experiments

All single-molecule imaging experiments were performed with a similar setting as described in our previous studies[3,26] and detailed below. In brief, the experiments were performed with a custom-built Nikon TI microscope equipped with a 100X/NA 1.49 oil-immersion TIRF objective (Nikon apochromat CFI Apo TIRF 100X Oil), an EMCCD camera (Andor iXon Ultra897), a perfect focus system to correct for axial drift and motorized laser illumination (Ti-TIRF, Nikon), and an incubation chamber maintaining a humidified 37 °C atmosphere with 5% CO_2 for the sample and the objective. Excitation was achieved using the following laser lines: 561 nm (1 W, Genesis Coherent) for JF549/PA-JF549 and TMR dyes; 633 nm (1 W, Genesis Coherent) for JF646/PA-JF646 dyes; 405 nm (140 mW, OBIS, Coherent) for all photo-activation experiments. Laser intensities were controlled by an acousto-optic Tunable Filter (AA Opto-Electronic, AOTFnC-VIS-TN) and triggered with the camera TTL exposure output signal. Lasers were directed to the microscope by an optical fiber, reflected using a multi-band dichroic (405 nm/488 nm/561 nm/633 nm quad-band, Semrock) and focused in the back focal plane of the objective. Emission light was filtered using single band-pass filters placed in front of the camera (Semrock 593/40 nm for TMR and JF549/PA-JF549 and Semrock 676/37 nm for JF646/PA-JF646). The angle of incident laser was adjusted for highly inclined laminated optical sheet (HiLo) conditions[27]. The microscope, cameras, and hardware were controlled through the NIS-Elements software (Nikon).

For ‘fast-tracking’ stroboscopic illumination (spaSPT) at ~133 Hz, the excitation laser (633 nm for PA-JF646 or 561 nm for PA-JF549) was pulsed for 1 ms at maximum (1 W) power at the beginning of the frame interval, while the photoactivation laser (405 nm) was pulsed during the

~447 μs camera transition time. Each frame consisted of a 7-ms camera exposure time followed by a ~447-μs camera inactive time. The camera was set for frame transfer mode and vertical shift speed at 0.9 μs. With this setup, the pixel size after magnification is 160 nm and the photon-to-grayscale gain is 109. Typically, 30000 frames with this sequence were collected per nucleus, during which the 405-nm intensity was manually tuned to maintain an average molecule density of ~0.5 localizations per frame, corresponding to ~15,000 localizations per cell per movie. Maintaining a very low density of molecules is necessary to avoid tracking errors.
For 'slow-tracking' (slow-SPT) experiments, long exposure times (50 ms, 100 ms, and 250 ms) and low constant illumination laser intensities (0.5% - 2% of 0.5 W power) were used to measure residence time. The camera was set for normal mode and vertical shift speed at 3.3 μs. We generally recorded each cell with 1200 frames for a 250 ms exposure time, 3000 frames for a 100 ms exposure time, or 6000 frames for a 50 ms exposure time. We included H2B-HaloTag cells for the photobleaching correction for each experiment.

spaSPT analysis
To estimate the likelihood of diffusion coefficients for each trajectory we used the SASPT package (v1.0) (https://github.com/alecheckert/saspt)[28]. The detailed discussion is available in Heckert et al[28]. In brief, we applied "State Array (SA)", a grid of state parameters that span a range of diffusion coefficients (0 to 100 μm^2/s), to calculate the posterior occupations of each point in the grid. The SA method conceptually produces a similar result as the Dirichlet process mixture models (DPMM) and retains its ability to model complex diffusive mixtures, while mitigating the issue of expensive likelihood functions. Instead of allowing infinite number of states ($K \rightarrow \infty$), the method fixes the number of states at a large but finite value. For each state $j = 1, \ldots, K$, the algorithm chooses a fixed set of state parameters θ_j. The model simplifies to:

$$\tau \sim \text{Direichlet}\left(\frac{\alpha}{K}, \ldots, \frac{\alpha}{K}\right)$$

$$Z_i \mid \tau \sim \text{Multinomial}\,(\tau, 1)$$

$$X_i \mid (Z_{ij} = 1) \sim f_X(x \mid \theta_j)$$

As a result, the SA method can compute more complex likelihood functions that incorporate localization error. Next, the algorithm infers the posterior p (Z, τ | X) with the Dirichlet prior $p(\tau)$ and corrects the systematic overestimation for the occupations of slow states due to defocalization. By marginalizing the posterior distribution on localization error, the method naturally incorporates uncertainty about the localization error of different states.
Alternatively, we analyzed the spaSPT data with the kinetic modeling framework implemented in the Spot-On package (v.1.0.4)[26]. Briefly, the model infers the diffusion constant and relative fractions of two or three subpopulations from the distribution of displacements computed at increasing lag times (1Δτ, 2Δτ,...). This is performed by fitting a semi-analytical model to the empirical histogram of displacements using non-linear least squares fitting. Defocalization is explicitly accounted for by modeling the fraction of particles that remain in focus over time as a function of their diffusion constant. We used the following setting for Spot-On analysis in the manuscript: `TimePoints = 8; BinWidth = 0.010; JumpsToConsider = 4; MaxJump = 5.05;`

```
ModelFit = CDF-fitting; NumberOfStates = 3; FitIterations = 3; FitLocErrorRange
= 0.010-0.075; LocError = 0.035; Dbound range = 0.0001-0.05; Dfree range =0.5-
25.
```

Slow-SPT analysis

To extract residence times from slow-SPT data, we used long exposure times (50 ms, 100 ms, or 250 ms) to motion-blur freely diffusing molecules into the background[3,29–31]. We then recorded the trajectory length of each 'bound' molecule and used these to generate a survival curve (1-CDF), and performed double-exponential fitting to estimate the unbinding rates for non-specific binding (K_{ns}) and specific binding (K_s). We note that localization errors can cause both false-positive and false-negative detections. The K_{ns} is likely to be contaminated by localization errors (e.g., from molecules close to being out-of-focus) and experimental noise. To filter out contributions from tracking errors and slow-diffusing molecules, we applied an objective threshold as previously described to consider only particles tracked for at least N_{min} frames. To determine N_{min}, we plotted the inferred residence time as a function of N_{min} and observed convergence to a single value after ~2.5 s. We thus used this threshold to determine the value of K_s. To correct the biases from photobleaching, cell drifting, and background fluctuating, we assume that all these factors should affect H2B-HaloTag to the same extent as those affecting YY1-HaloTag. We can use an apparent unbinding rate for H2B-HaloTag as K_{bias}, consistent with our FRAP analysis. Thus, we performed the slow-SPT experiments for YY1-HaloTag and H2B-HaloTag with the same camera and laser settings on the same day. We then obtained the residence time as:

$$\tau_{\mathrm{s}} = \frac{1}{k_s - k_{bias}} = \frac{1}{k_{\mathrm{s,\,true}}}$$

FRAP image analysis

To quantify FRAP movies, we wrote a pipeline in MATLAB (v2021a). Briefly, our algorithm automatically detects the bleached spot, the background spot, and the nucleus segments by Gaussian smoothing, hole-filling, and segmenting a nucleus in a FRAP movie. Cell drift is also automatically corrected by the optimal linear translation in x and y. Next, we quantify the bleach spot signal as the mean intensity of a slightly smaller region, which is more robust to lateral drift. The FRAP signal is corrected for photobleaching using the measured reduction in total nuclear fluorescence and internally normalized to its mean value during the 20 frames before bleaching. Finally, corrected FRAP curves from each single cell were averaged to generate a mean FRAP recovery. We used the mean FRAP recovery in all figures and for model-fitting.

Model selection is critical to infer the parameters from FRAP experiments. Sprague et al.[32] suggested that when:

$$\frac{k_{\mathrm{ON}}^{*} w^2}{D_{\mathrm{FREE}}} \ll 1 \text{ and } \frac{\mathrm{k_{OFF}}}{\mathrm{k_{ON}^{*}}} \lesssim 1$$

Then a 'reaction dominant' FRAP model is most appropriate. For YY1:

$$\frac{\mathrm{k_{ON}^{*} w^2}}{\mathrm{D_{FREE}}} = 0.00256 \ll 1$$

Thus, a reaction-dominant FRAP model is the most suitable choice for YY1's FRAP modeling. Sprague et al.[32] demonstrated that the FRAP recovery depends only on k_{OFF} in the reaction-dominant regime. We thus fit the FRAP curves to the model and applied the slower off rate to estimate the residence time according to $\tau s = \frac{1}{k_{off}}$.

Inferring parameters related to YY1's target search mechanism

We used the parameters inferred from our spaSPT and the residence time measurements from our FRAP or slow-SPT analysis. The detailed discussion is available in Hansen et al[3]. Briefly, from the Spagl State Array analysis, we determined that the total bound fraction for YY1 is ~31%. However, the total bound fraction (0 – 0.1 μm^2/s) contains both YY1 molecules bound specifically to their target sites and non-specific interactions (e.g., sliding or jumping on DNA). We previously estimated the fraction that is non-specifically bound using a mutant CTCF with a His-to-Arg mutation in each of the 11 zinc-finger domains[3]. This CTCF mutant is virtually unable to interact specifically with its binding sites. The Spagl analysis estimated the bound fraction to be ~8.1% for this mutant in mESCs. Since we did not perform the spaSPT experiments for YY1's DNA binding domain mutants, we thus inferred the $F_{BOUND,specifc}$ ~= $F_{BOUND,total}$ in this manuscript.

We next determined the average time for YY1 to find its cognate site after dissociating from the previous site. We will use '*s*' and '*ns*', as abbreviations for specific and non-specific binding, respectively, in the following discussion. The pseudo-first-order rate constant for specific binding sites, $k^*_{ON,s}$, is related to the fraction bound by:

$$F_{\mathrm{BOUND,S}} = \frac{k^*_{\mathrm{ON,s}}}{k^*_{\mathrm{ON,s}} + k^*_{\mathrm{OFF,s}}} \Leftrightarrow k^*_{\mathrm{ON,s}} = \frac{F_{\mathrm{BOUND,s}} k_{\mathrm{OFF,s}}}{1 - F_{\mathrm{BOUND,s}}}$$

We determined the off-rate for a specific interaction in our residence time measurements. Thus, we can calculate $k^*_{ON,s}$, which is directly related to the average search time for a specific YY1-binding site:

$$\tau_{\mathrm{search}\ ,s} = \frac{1}{k^*_{\mathrm{ON,s}}} = \frac{1 - F_{\mathrm{BOUND,s}}}{F_{\mathrm{BOUND,s}} k_{\mathrm{OFF,s}}}$$

After plugging in these determined parameters of $F_{BOUND,s}$ and $k_{OFF,s}$, we obtained total search times for YY1 of ~28.3 s in wild type mES cells, ~31.6 s in CTCF-depleted cells, ~61.4 s in RAD21-depleted cells. We inferred the residence time estimated from the slow-SPT data with 100 ms exposure time or the FRAP analysis in this manuscript.

Supplementary Methods References

1. Ran, F. A. *et al.* Genome engineering using the CRISPR-Cas9 system. *Nat Protoc* **8**, 2281–2308 (2013).

2. Concordet, J.-P. & Haeussler, M. CRISPOR: intuitive guide selection for CRISPR/Cas9 genome editing experiments and screens. *Nucleic Acids Res* **46**, gky354- (2018).

3. Hansen, A. S., Pustova, I., Cattoglio, C., Tjian, R. & Darzacq, X. CTCF and cohesin regulate chromatin loop stability with distinct dynamics. *Elife* **6**, e25776 (2017).

4. Gabriele, M. *et al.* Dynamics of CTCF- and cohesin-mediated chromatin looping revealed by live-cell imaging. *Science* **376**, 496–501 (2022).

5. Natsume, T., Kiyomitsu, T., Saga, Y. & Kanemaki, M. T. Rapid Protein Depletion in Human Cells by Auxin-Inducible Degron Tagging with Short Homology Donors. *Cell Reports* **15**, 210–218 (2016).

6. Li, S., Prasanna, X., Salo, V. T., Vattulainen, I. & Ikonen, E. An efficient auxin-inducible degron system with low basal degradation in human cells. *Nat Methods* **16**, 866–869 (2019).

7. Testa, A. *et al.* Chromatin Immunoprecipitation (ChIP) on Chip Experiments Uncover a Widespread Distribution of NF-Y Binding CCAAT Sites Outside of Core Promoters*. *J Biol Chem* **280**, 13606–13615 (2005).

8. Langmead, B. & Salzberg, S. L. Fast gapped-read alignment with Bowtie 2. *Nat Methods* **9**, 357–359 (2012).

9. Zhang, Y. *et al.* Model-based Analysis of ChIP-Seq (MACS). *Genome Biol* **9**, R137 (2008).

10. Ramírez, F. *et al.* deepTools2: a next generation web server for deep-sequencing data analysis. *Nucleic Acids Res* **44**, W160–W165 (2016).

11. Tu, S. *et al.* MAnorm2 for quantitatively comparing groups of ChIP-seq samples. *Genome Res* **31**, 131–145 (2020).

12. Hsieh, T.-H. S. *et al.* Resolving the 3D Landscape of Transcription-Linked Mammalian Chromatin Folding. *Mol Cell* **78**, 539-553.e8 (2020).

13. Pettitt, S. J. *et al.* Agouti C57BL/6N embryonic stem cells for mouse genetic resources. *Nat Methods* **6**, 493–495 (2009).

14. Servant, N. *et al.* HiC-Pro: an optimized and flexible pipeline for Hi-C data processing. *Genome Biol* **16**, 259 (2015).

15. Yardımcı, G. G. *et al.* Measuring the reproducibility and quality of Hi-C data. *Genome Biol* **20**, 57 (2019).

16. Sauria, M. E. & Taylor, J. QuASAR: Quality Assessment of Spatial Arrangement Reproducibility in Hi-C Data. *Biorxiv* 204438 (2017) doi:10.1101/204438.

17. Ursu, O. *et al.* GenomeDISCO: a concordance score for chromosome conformation capture experiments using random walks on contact map graphs. *Bioinformatics* **34**, 2701–2707 (2018).

18. Durand, N. C. *et al.* Juicer Provides a One-Click System for Analyzing Loop-Resolution Hi-C Experiments. *Cell Syst* **3**, 95–98 (2016).

19. Crane, E. *et al.* Condensin-driven remodelling of X chromosome topology during dosage compensation. *Nature* **523**, 240–4 (2015).

20. Rao, S. S. P. *et al.* A 3D Map of the Human Genome at Kilobase Resolution Reveals Principles of Chromatin Looping. *Cell* **159**, 1665–1680 (2014).

21. Flyamer, I. M., Illingworth, R. S. & Bickmore, W. A. Coolpup.py: versatile pile-up analysis of Hi-C data. *Bioinformatics* **36**, 2980–2985 (2020).

22. Ardakany, A. R., Gezer, H. T., Lonardi, S. & Ay, F. Mustache: multi-scale detection of chromatin loops from Hi-C and Micro-C maps using scale-space representation. *Genome Biol* **21**, 256 (2020).

23. Matthey-Doret, C. *et al.* Computer vision for pattern detection in chromosome contact maps. *Nat Commun* **11**, 5795 (2020).

24. Quinlan, A. R. & Hall, I. M. BEDTools: a flexible suite of utilities for comparing genomic features. *Bioinformatics* **26**, 841–842 (2010).

25. Nojima, T., Gomes, T., Carmo-Fonseca, M. & Proudfoot, N. J. Mammalian NET-seq analysis defines nascent RNA profiles and associated RNA processing genome-wide. *Nat Protoc* **11**, 413–428 (2016).

26. Hansen, A. S. *et al.* Robust model-based analysis of single-particle tracking experiments with Spot-On. *Elife* **7**, e33125 (2018).

27. Tokunaga, M., Imamoto, N. & Sakata-Sogawa, K. Highly inclined thin illumination enables clear single-molecule imaging in cells. *Nat Methods* **5**, 159–161 (2008).

28. Heckert, A., Dahal, L., Tjian, R. & Darzacq, X. Recovering mixtures of fast diffusing states from short single particle trajectories. *Biorxiv* 2021.05.03.442482 (2021) doi:10.1101/2021.05.03.442482.

29. Sergé, A., Bertaux, N., Rigneault, H. & Marguet, D. Dynamic multiple-target tracing to probe spatiotemporal cartography of cell membranes. *Nat Methods* **5**, 687–694 (2008).

30. Chen, J. *et al.* Single-molecule dynamics of enhanceosome assembly in embryonic stem cells. *Cell* **156**, 1274–85 (2014).

31. Mazza, D., Abernathy, A., Golob, N., Morisaki, T. & McNally, J. G. A benchmark for chromatin binding measurements in live cells. *Nucleic Acids Res* **40**, e119–e119 (2012).

32. Sprague, B. L., Pego, R. L., Stavreva, D. A. & McNally, J. G. Analysis of Binding Reactions by Fluorescence Recovery after Photobleaching. *Biophys J* **86**, 3473–3495 (2004).

Supplementary Notes

I. Technical controls of Micro-C assay

To address if the fine-scale chromatin loops/dots detected in Micro-C are due to higher accessibility of micrococcal nuclease (MNase) to promoters and enhancers, we here cite other studies and present results of additional analyses on our Micro-C data that demonstrate accessibility biases are largely negligible.

1. Micro-C data normalization

The 3C-based assays involve a series of biochemical reactions that may introduce noise to the data analysis and the final results. Such noise must be eliminated before interpreting the data. Early normalization methods for Hi-C data focused on explicit factors causing noise. Factors like sequence uniqueness, GC content, uneven distribution of restriction sites across the genome may introduce biases into the 3C-based analysis[1]. As a solution to these issues, the Mirny and Aiden labs proposed using matrix balancing methods to handle all noise sources "implicitly"[2,3]. Two fundamental assumptions underlie the methods: 1) visibility across all genomic regions should be equal, and 2) all Hi-C biases are one-dimensional and factorizable. A balanced matrix should allow equal visibility to any genomic locus regardless of system biases, that is, a normalized matrix whose rows and columns sum to the same amount. In Micro-C/Hi-C analysis, three primary matrix balancing algorithms are commonly used, including "the square root of vanilla coverage (VC)", "Knight-Ruiz (KR)", and "Iterative Correction and Eigenvector decomposition (ICE)". The idea of VC is similar to coverage normalization as it divides each matrix element by its row sum and column sum to eliminate different sequencing coverage of each locus. ICE and KR repeat the VC process until all rows and cols sum to the same value and converge. Results from KR and ICE are typically nearly identical and highly reproducible. Additionally, we often performed distance normalization (e.g., observed/expected) to remove the bias caused by 1D distance-dependent interactions in 3C-based data.

2. MNase digests chromatin uniformly

Recent studies from the Henikoff and Längst labs have experimentally and computationally addressed this issue[4,5], indicating that MNase equally accesses DNaseI-hypersensitive and non-hypersensitive sites, sites of active and inactive transcription, and euchromatin as well as heterochromatin (see Fig.9 in Chereji et al, Fig.4 in Schwartz et al, and a snapshot from Chereji et al in **Supplementary Fig. 1** below). They also state that "*although heterochromatin and euchromatin appear different when observed cytologically at low resolution, at the molecular level, MNase and other proteins can access heterochromatin regions at rates similar to those of accessing euchromatin*". We thus conclude that MNase can digest chromatin uniformly across the genome regardless of its accessibility level.

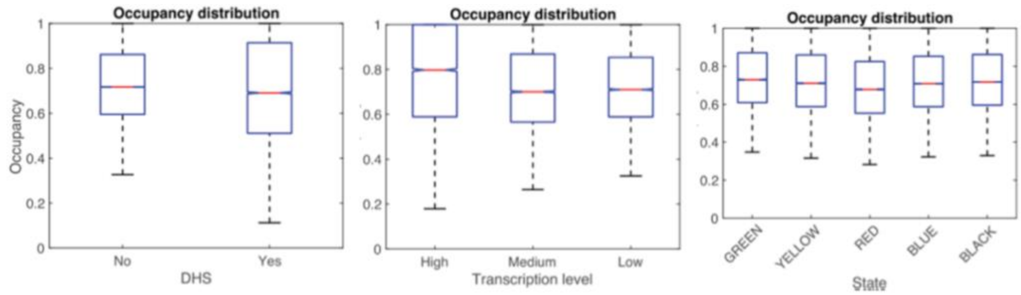

Supplementary Fig. 1. Nucleosome occupancy mapped in *Drosophila* S2 cells by MNase-seq. Rightmost panel: green (HP1-bound), yellow (active), red (active), blue (Polycomb-bound) and black (repressive) chromatin states defined in[78]

3. Micro-C is not biased toward chromatin accessible regions

Previously, we and others have independently demonstrated that Micro-C contacts are not biased toward highly accessible chromatin regions[6–8]. Notably, a recent Micro-C-based technique, Micro-Capture-C (MCC), that focuses on the interactions around promoter regions also did not find substantial bias toward DNaseI hypersensitive sites[8]. To further support this point, we provide key results below demonstrating that Micro-C data is not affected by chromatin accessibility levels. We thus conclude that the E-P interactions captured by Micro-C represent *bona fide* proximal chromatin contacts.

- When we analyzed Micro-C reads as single-end data like ChIP-seq or MNase-seq, we do not observe any apparent correlation with chromatin accessibility. In **Supplementary Fig. 2, panel i**, we show that neither raw ($r^2 = 0.12$) nor balanced ($r^2 << 0.1$) Micro-C data show substantial correlation with ATAC-seq signal. While the raw Micro-C data shows an anti-correlation to ATAC-seq around ATAC-seq peaks, this trend disappears after matrix balance (**Supplementary Fig. 2, panel ii**). We note that all the Micro-C data in this manuscript were normalized either by matrix balancing (detailed in the Methods section).
- If contacts at accessible promoters/enhancers were artifactually amplified by Micro-C, we should expect them to be far more prevalent than contacts at polycomb/heterochromatin regions. However, genome-wide *P(s)* analysis shows no evidence of significant differences in contact probability for different types of chromatin regions, including accessible promoters and enhancers, Polycomb-bound and heterochromatic domains, within a 500-kb range (**Supplementary Fig. 2, panel iii**).
- As we showed in our previous work[6], very deeply sequenced Hi-C data[9] can recapitulate some E-P loops detected by Micro-C (**Supplementary Fig. 2, panel iv**). While these dots in Hi-C are much fuzzier and their precise locations are often indiscernible, the example below clearly indicates that they are present in both data. It is widely accepted in the 3D-genome field that Hi-C has minimal bias towards highly accessible chromatin, so contact enrichment around E-P interactions present in both methods cannot be an artifact of Micro-C. Critically, Micro-C can further resolve what appears as a large blurry dot in Hi-C into multiple finer-scale dots and pinpoint the precise location of their interactions (**Supplementary Fig. 2, panel iv**, zoomed-in box), indicating that Micro-C is a superior method to Hi-C in identifying fine-scale interactions.

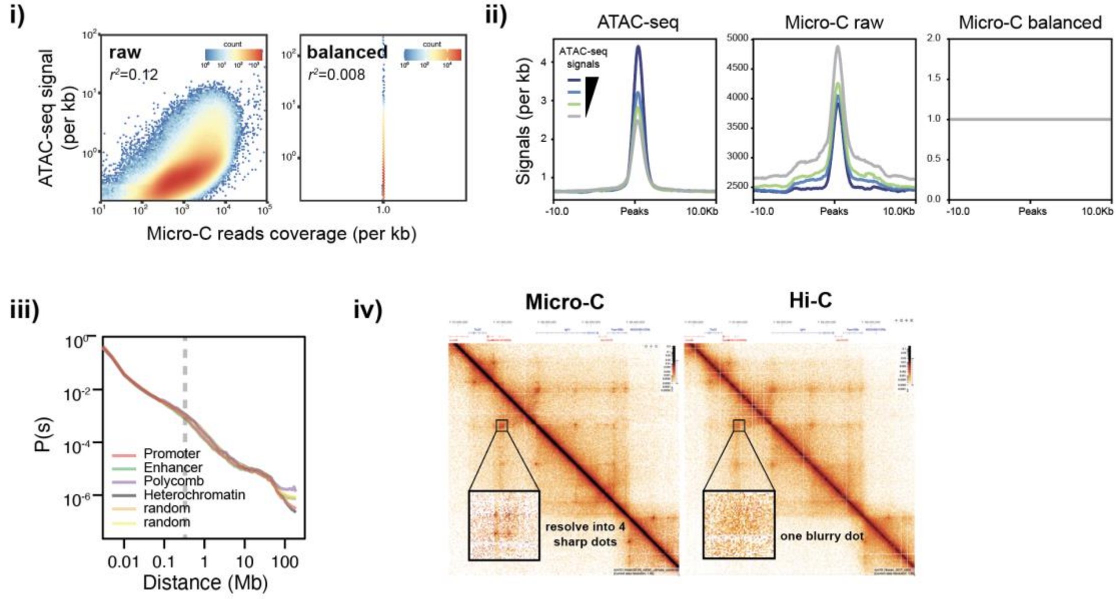

Supplementary Fig. 2. Quality control of Micro-C analysis. i) Scatter plots with density heatmap compare the correlation between Micro-C reads coverage per kb (x-axis) and ATAC-seq signal per kb (y-axis). Neither raw Micro-C data (r^2 = 0.11) nor the balanced data (r^2 << 0.01) show substantial correlation with ATAC-seq signal. ii) Histogram shows the genome-wide averaged signal enrichment of ATAC-seq (left), Micro-C raw signals (middle), or Micro-C balanced signals across a ±3-kb region. Data are grouped by the intensity of ATAC-seq from high to low. Raw Micro-C signals are slightly higher in regions with low ATAC-seq signals than in regions with high ATAC-seq signals. The balanced data shows homogeneous coverage throughout the genome. iii) Genome-wide contact decaying P(s) analysis across the regions enriched with different chromatin features, including promoter, enhancer, polycomb, heterochromatin, and random loci. iv) Snapshots of Micro-C vs. Hi-C around *Igf1r* region.

4. 1D coverage does not explicitly affect loop calling in the high-resolution data

To test whether loop calling is affected by coverage in our data, we analyzed "1D coverage vs. the number of significant loops per locus". **Supplementary Fig. 3** below shows a similar trend across all resolutions, suggesting that 1D coverage does not explicitly affect dot calling in the high-resolution data (Wilcoxon Rank Sum and Signed Rank Tests). We also included the balanced data that demonstrates the number of loops is not dependent on the coverage.

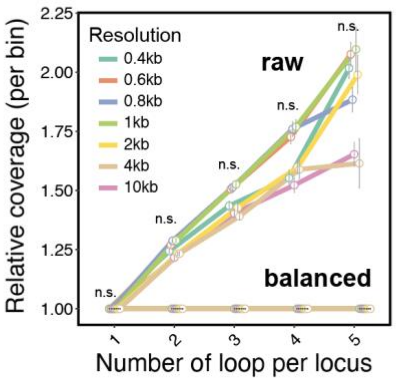

Supplementary Fig. 3. Quality control of loop calling with Micro-C data (raw and balanced signals) at different resolutions. The curves show the relationship between the number of loops being identified per locus (x-axis) and Micro-C reads coverage (y-axis; Top group=raw; Bottom group=balanced). A similar trend across all resolutions suggests that 1D coverage does not explicitly affect loop calling in the high-resolution data (Wilcoxon Rank Sum and Signed Rank Tests; n=37 biologically samples; Data are presented as mean values ±SD).

5. Micro-C analysis is not biased by global changes in chromatin interactions

To test whether these normalization approaches can address global changes in genome organization and correct systemic biases, we computationally injected 3x more long-range interactions (> 2Mb) and inter-chromosomal interactions into wild-type Micro-C data (**Supplementary Fig. 4**, top panel). In this case, the artificially injected reads generated an extreme case of reads being redistributed to longer ranges (**Supplementary Fig. 4**, top panel). Importantly, the results of compartment strength, TADs, cohesin loops, and E-P/P-P loops are not affected by the global changes caused by artificial reads (**Supplementary Fig. 4**, bottom panels). We therefore conclude that matrix balancing and distance normalization can reveal the bona fide chromatin structures and differential chromatin interactions regardless of systemic biases and global changes.

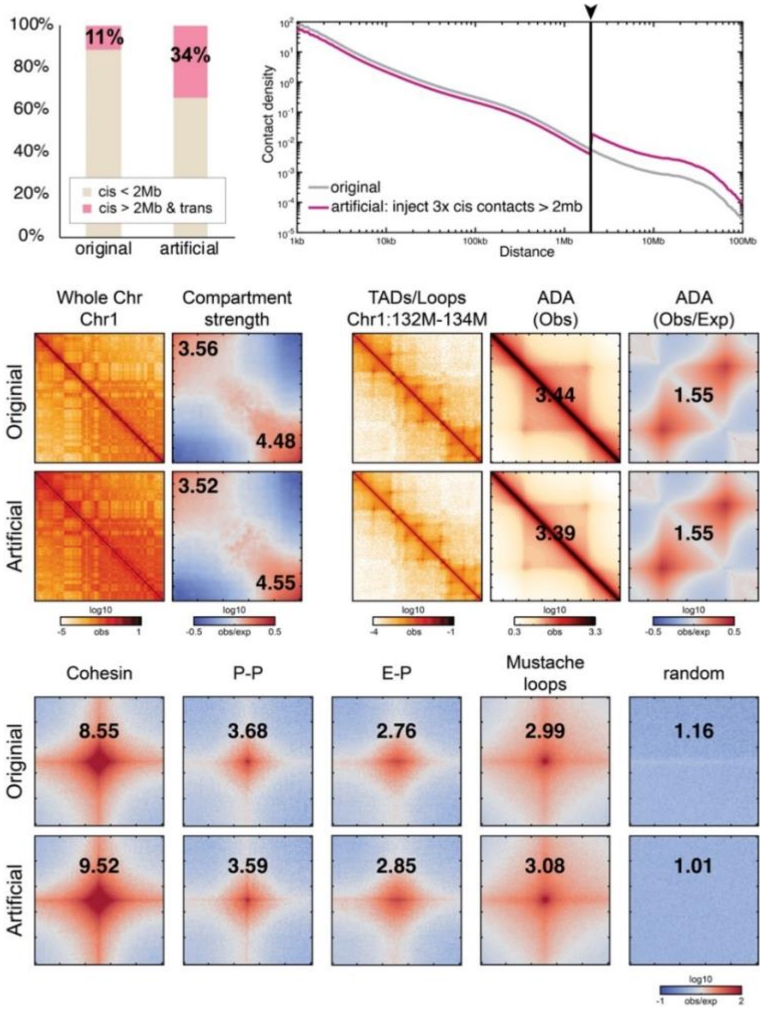

Supplementary Fig. 4. Micro-C analysis is not biased by global changes in chromatin interactions.

II. Technical controls of auxin-inducible degradation

1. The effect of basal degradation due to degron leakiness

To determine to what extent degron leakiness may affect our "untreated" cells, we quantified CTCF, RAD21, WAPL, and YY1 protein levels in each degron clone and compared them to wild-type cells by Western blot (**Extended Data Fig. 2b-c**). We also conducted correlation test, reproducibility tests and differential analyses of all untreated samples in ChIP-seq (**Extended Data Fig. 3a-c**), Micro-C (**Extended Data Fig. 4a-d**), and RNA-seq data (**Extended Data Fig. 5a**). Although CTCF-AID, RAD21-AID, and YY1-AID cell lines show some basal degradation (~40%, ~30%, ~25% reduction of protein levels compared to wild type cells, respectively), we find no significant changes in chromatin association, 3D genome organization, or transcriptome in these untreated cells. The results of our quality controls are summarized below in **Supplementary Fig. 5a**.

Consistent with differential expression analysis identifying only a few significantly altered genes after 3-hour depletion, Principal component analysis (PCA) of RNA-seq and mNET-seq revealed that all the clonal cell lines derived from the C59 parental clone (see Methods for details; control (C59), ΔCTCF (C58), ΔRAD21 (F1), ΔWAPL (C40) clones) with or without IAA treatment are clustered together, except for cells depleted of RAD21 after 12 and 24 hours (DEGs > 1000). Additionally, we found that YY1-depleted cells are clustered separately from C59-derived cells and are more similar to wild-type JM8.N4 cells. The results of PCA are summarized below in **Supplementary Fig. 5b**.

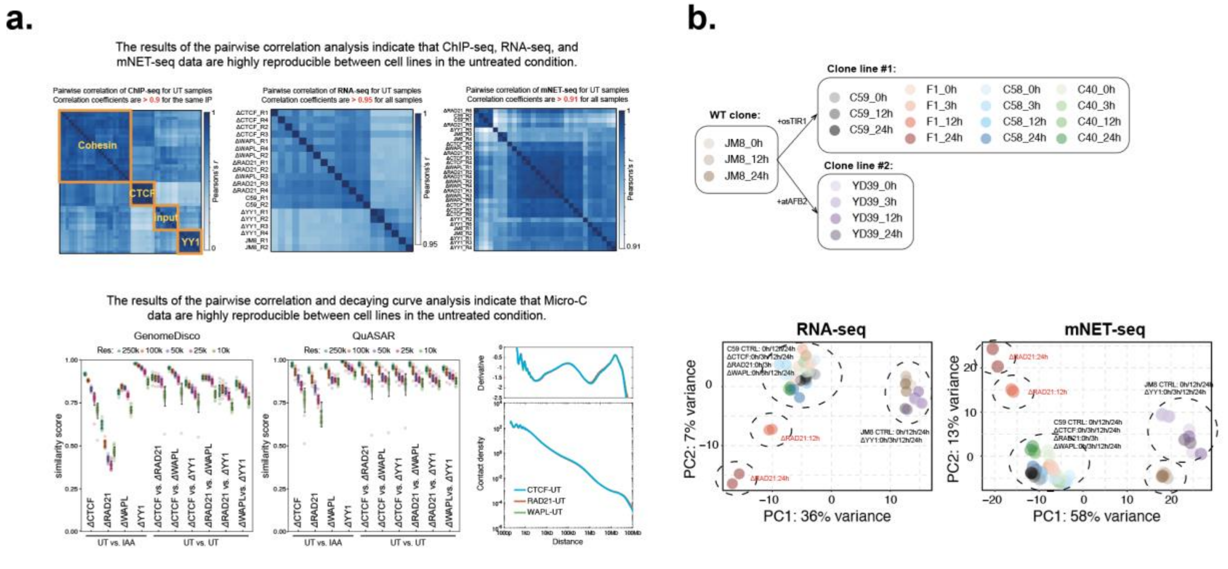

Supplementary Fig. 5. a) The effect of basal degradation due to degron leakiness. b) Principal component analysis of RNA-seq (left panel) or mNET-seq (right panel) shows the separation of samples. X-axis: the scores of the first component (PC1). Y-axis: the scores of the second component (PC2). Cell lines derived from the C59 parental clone (C59, ΔCTCF, ΔRAD21, or ΔWAPL cell lines) are clustered together, except for 12- and 24-hour ΔRAD21 cells. We note that ΔYY1 cell lines derived from another clone expressing AtAFB2 protein are clustered separately and more similar to the wild-type JM8.N4 cells.

2. The effect of CTCF, cohesin, and WAPL depletion on cell cycle and cell death

Consistent with previous reports[10,11], CTCF or WAPL depletion does not cause cell cycle abnormalities and cell death within 24 hours of treatment with indole-3-acetic acid (IAA) (**Supplementary Fig. 6**), while RAD21 depletion leads to substantial cell cycle arrest at G2 and cell death within 6 hours of degradation (**Supplementary Fig. 6**).

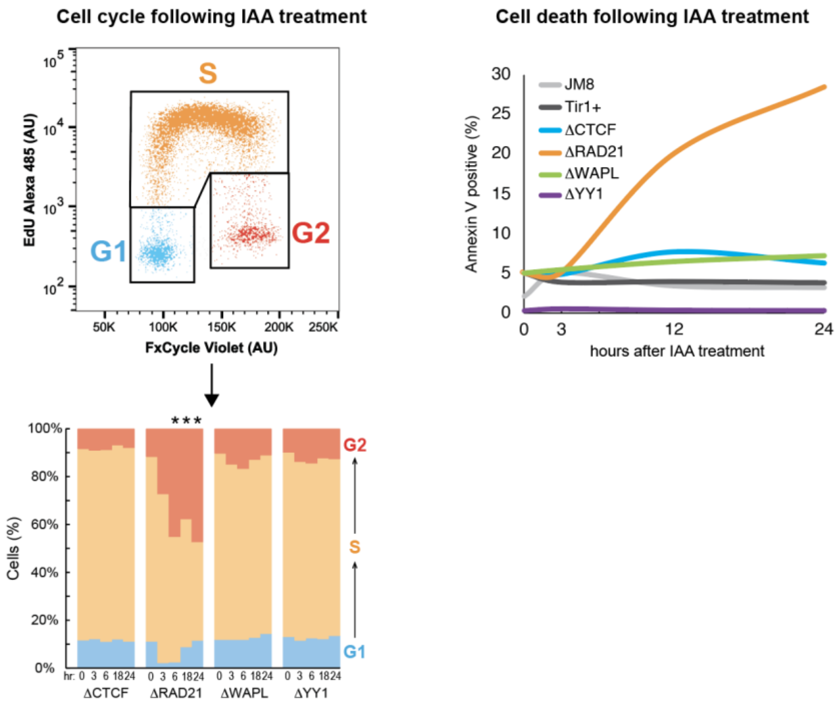

Supplementary Fig. 6. Cell cycle and death analysis. (Left) Representative cell cycle distribution is shown in the top panel. G1 and G2/S phases are separated by FxCycle violet staining. G1/G2 (x-axis) and S phases are separated by EdU Alexa 488 staining (y-axis). Bar graph shows cell cycle distribution across the degradation time course for parental and target-depleted cell lines. (Right) Curves show the cell death rate in wild-type, parental, and target-depleted cells across a degradation time course. Statistical test: Two-sided t-test compared to time=0. *: p-value < 0.01

3. Summary of RNA-seq and nascent RNA-seq from previous studies and this study

Our degradation time course revealed that CTCF depletion leads to immediate transcriptional deregulation of ~60 genes (p_{adj} < 0.01 & > 2-fold change) (**Fig. 3h** and **Extended Data Fig. 5c-d**). The result is consistent with a PRO-seq analysis [12] that reported ~122 DEGs (p_{adj} < 0.05 & > 2-fold change) after 4 hours of CTCF depletion. While RAD21 depletion for 3 hours did not cause significant transcriptional changes, extended cohesin depletion led to drastic transcriptional

deregulation (over thousands of DEGs), possibly because of the secondary effects such as cell cycle defects (**Fig. 3h** and **Extended Data Fig. 5c-d**). While our WAPL depletion clearly increases the fraction of cohesin retention on chromatin (~35%) (**Extended Data Fig. 2f**) and expands the grid of cohesin loops in Micro-C maps (**Extended Data Fig. 4h**), we do not detect a global effect on transcription across the time course (~10 DEGs, $p_{adj} < 0.01$ & > 2-fold change) (**Extended Data Fig. 5c-d**), contradicting the previous study that found ~779 DEGs ($p_{adj} < 0.05$) after 6-hour WAPL depletion using TT-seq[11]. We noticed that we have used a more stringent cut-off to call DEGs than other studies. By adjusting the threshold to $p_{adj} < 0.05$, we can detect more DEGs in the range comparable to previous studies (DEGs=~394).

Together, we conclude that 1) our mNET-seq method is sensitive to massive transcriptional alterations; 2) our mNET-seq results largely agree with previous reports; and 3) many of the DEGs detected using "$p_{adj} < 0.05$" fail to survive with "$p_{adj} < 0.01$ & 2-fold change", indicating that these changes to gene expression are mild (see Table below).

mNET-seq or similar nascent RNA profiling methods

Depletion	Time after +IAA / previous studies	padj < 0.01 shrinkage(2x FC)	padj < 0.01 2x FC	padj < 0.01	padj < 0.05
CTCF	3h	50	73	212	295
	12h	69	117	327	556
	24h	76	194	646	1183
	Luan et al, G1E-ER4, 4h, PRO-seq				122
RAD21	3h	5	6	94	196
	12h	941	1313	3754	5240
	24h	2274	2727	5353	7680
	Thiecke et al, HeLa, 1h, SLAM-seq				687
	Rao et al, HCT-116, 6h, PRO-seq				4196
WAPL	3h	2	2	5	6
	12h	9	39	147	394
	24h	11	44	164	437
	Liu et al 2021, 6h, TT-seq				779
	Liu et al 2021, 24h, TT-seq				2733

* Data in this study are colored with darker shade.
* This manuscript reports the results obtained with the most stringent cut-off ($p_{adj} < 0.01$ & > 2-fold change after LFC shrinkage).

III. Additional ChIP-seq analysis compared to previous studies

1. Proteins that enriched at Enhancer, promoter, Cohesin loop anchors

We plotted ChIP-seq data for various histone marks, TFs, and chromatin remodelers over the major types of loop anchors (**Supplementary Fig. 7**). Consistent with our previous characterization of the subtypes of chromatin structures below the scale of TADs[6] as well as a recent imaging study[13], loop anchors enriched in CTCF and cohesin generally do not colocalize with sites of active transcription. In contrast, various TFs, coactivators, and Pol II are associated with E-P and P-P anchors.

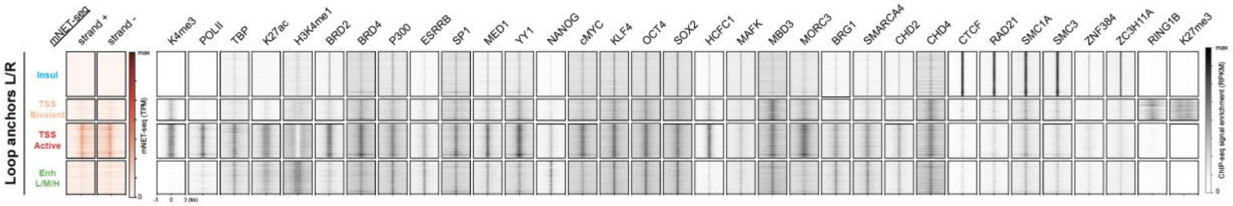

Supplementary Fig. 7. Heatmaps of mNET-seq and ChIP-seq signal enrichments in a · 3-kb window around the four primary types of loop anchors.

2. Cohesin occupancy at active promoters

It has been reported that cohesin is also positioned by actively transcribing Pol II and by the transcription machinery[14,15] but the ChIP signal at these putative "CTCF-independent" loci is generally quite weak[16] (**Supplementary Fig. 8a**). Specifically, promoter-bound cohesin peaks were barely detectable in untreated cells, and though they slightly increased after CTCF depletion, we could still only detect a few thousand upregulated cohesin peaks upon CTCF degradation (**Supplementary Fig. 8a-b**)[14]. We thus suggest that in the absence of CTCF, the transcription machinery may halt cohesin extrusion but not as effectively as CTCF does. Whether the promoter-bound cohesin peaks detected after CTCF depletion are functional remains to be determined.

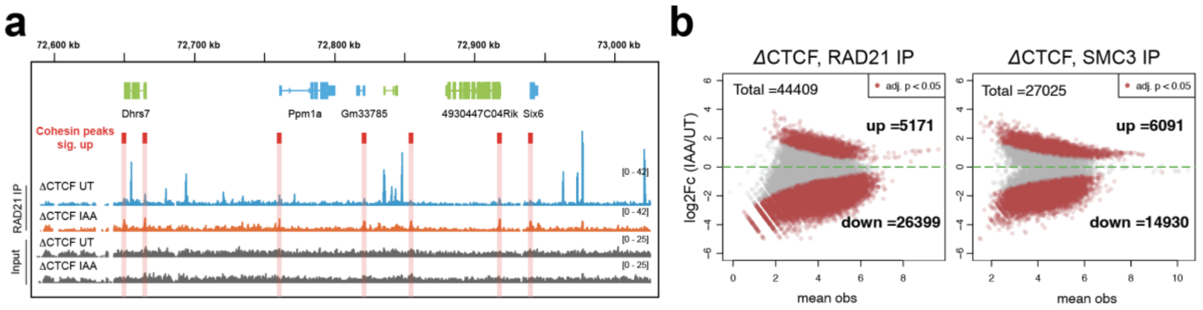

Supplementary Fig. 8. a) Snapshot of an example showing the position and level of cohesin at promoters (red shade) after CTCF degradation. b) MA plots showing the differential ChIP-seq peaks of RAD21 and SMC3 in the CTCF-depleted cells. The differential peaks (adjusted p-value < 0.05) are colored in red. X-axis: mean observations of UT and IAA cells. Y-axis: log2 fold-change comparing the UT and IAA-treated cells.

3. WAPL and cohesin occupancy near SOX2/OCT4 binding sites

WAPL depletion leads to cohesin retention on chromatin[17], appreciable both by ChIP-seq (**Fig. 3c-e**, **Extended Data Fig. 3e-g**) and by salt fractionation, (**Extended Data Fig. 2f**, orange box). A recent study reported that thousands of cohesin peaks are lost near SOX2 and OCT4 binding sites after WAPL depletion[11]. Nonetheless, we did not observe such change (**Supplementary Fig. 9**), perhaps due to differences in WAPL degradation levels or the duration of depletion. Taken together, our ChIP-seq data confirmed effective degradation of loop extrusion factors within 3 hours and largely recapitulated previous observations[10,16,18].

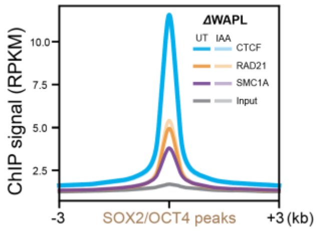

Supplementary Fig. 9. Profiles of genome-wide averaged ChIP-seq for CTCF, RAD21, SMC1A, and input around the ・ 3-kb region of SOX2 or OCT4 peaks in WAPL-depleted cells.

IV. Additional notes and analysis of chromatin loops

1. Loop extrusion model

Elegant experiments combining acute protein depletion of CTCF, cohesin, and cohesin regulatory proteins with Hi-C or imaging approaches have revealed the role of CTCF and cohesin in regulating the first two levels, TADs and compartments[10,18–21]. These studies have shown that while CTCF plays only a minor role in compartmentalization, CTCF and cohesin removal largely eliminates TADs and chromatin loops anchored by these proteins across the genome. CTCF and cohesin are thought to form TADs and loops through DNA loop extrusion[22,23]. This model posits that cohesin extrudes bidirectional loops until it encounters convergent and occupied CTCF binding sites. When averaged across cell populations, the extruded chromatin appears to be spatially organized into a self-interacting domain (TAD or loop domain), and CTCF binding sites constitute the domain boundaries that restrict inter-domain interactions. Halting of cohesin extrusion at CTCF sites sometimes gives rise to sharp corner peaks in contact maps, known as loops or corner dots. WAPL, a cohesin unloader, releases cohesin from chromatin and WAPL depletion therefore increases cohesin residence times as well as the amount of cohesin on chromatin[19,21].

2. Example of the consequences of perturbing CTCF/Cohesin

Paradigmatic experiments focusing on mouse development suggested that TAD disruption through inversion or deletion of CTCF sites around developmental genes such as *Epha4*, *Kcnj*, or *Ihh* can cause severe limb malformation[24]. Similar studies at the locus encoding the morphogen Sonic hedgehog have led to somewhat inconsistent effects on transcription and developmental phenotypes, perhaps due to manipulation of different CTCF sites[25,26]. Furthermore, CTCF and cohesin appeared to be crucial to some biological processes such as neuronal maturation[27] and lipopolysaccharide-induced inflammatory response[28], but their presence seemed dispensable in other cases such as neuronal activity-dependent transcription[27] and immune cell transdifferentiation[28].

3. E-P/P-P interacts across TAD boundaries

Our data reporting that ~30% of loops cross a TAD boundary are largely consistent with previous studies. First, a recent CRISPRi-based enhancer screening reported that ~30% of E-P interactions

do not fall into the same TAD[29]. Second, promoter Capture Hi-C experiments[30] also observed that ~24% of promoter-centered interactions are not constrained within the same TAD, which aligns with our findings. Third, emerging evidence by single-cell Hi-C and super-resolution imaging indicates that TADs are dynamic chromatin structures that constantly form and dissolve[31–35]. Associations between regions in the same TAD are not necessarily pervasive and interactions are common between neighboring TADs[36,37]. Cohesin can traverse across boundaries frequently and mediate domain intermingling[38,39]. Fourth, the latest evidence indicates that the perturbations of TAD boundaries around the Sox2 and HoxD regions are mostly harmless to embryo development[40,41], suggesting that high affinity enhancer-promoter interactions can overcome structural barriers. Finally, since the eQTL analysis in Delaneau et al., is based on the correlation of a set of ChIP-seq data for histone modifications, the cis-regulatory domains (CRDs) identified in this study most likely represent local chromatin domains segregated by chromatin states (e.g., active or inactive regions) rather than long-range E-P loops. It is uncertain whether this computational structure fully reflects 3D genome structures in situ. A recent study indicates that analysis of chromatin loops outperforms eQTL in explaining neurological GWAS results, suggesting there may be much ampler information in high-resolution 3D genome maps than in eQTL analyses[42]. Together, our findings of cross-TAD interactions are largely consistent with other studies, arguing that communication between enhancers and their cognate genes in another domain is not uncommon.

4. What are the 10-20% CTCF/cohesin-sensitive E-P/P-P loops?

Previous studies reported that CTCF and cohesin appear to mediate the long-range E-P loops but have a lesser impact on short-range loops[43]. Our genome-wide analysis reveals that long-range chromatin loops are more susceptible to disruption (**Extended Data Fig. 6g**), with CTCF/cohesin-sensitive loops being much larger (median = ~85 kb for E-P and ~102 kb for P-P) than the insensitive loops (median = ~41 kb for E-P and ~71 kb for P-P). According to the loop extrusion model, cohesin might either directly bridge E-P interactions at TAD boundaries, or the process of extrusion might inherently increase the frequency of long-range E-P interactions[44]. To test this possibility, we compared the genomic features of the CTCF/cohesin-sensitive loops relative to TAD boundaries. CTCF and cohesin occupancy at the anchors of these loops is much higher than at the unaffected loop anchors (**Extended Data Fig. 6h**), suggesting that these loops may overlap with TAD boundaries, where CTCF and cohesin proteins are highly enriched. However, only ~5% of the CTCF/cohesin-sensitive loop anchors are located within a 1-kb window surrounding TAD boundaries (**Fig. 4f-g**). In fact, most of them are located even farther away than the unaffected loops (**Fig. 4f-g** and **Extended Data Fig. 6i**) and tend to interact with another DNA locus within the same TAD without crossing the boundary (**Fig. 4g)**.

5. Loop at specific CTCF sites

Interestingly, in comparison to a recent finding[12], our data show only moderate CTCF persistence at architectural sites (e.g., loop anchors or TAD borders) upon CTCF degradation (**Supplementary Fig. 10a**). Interactions anchored by CTCF binding sites associated with mitotic bookmarking[45]

and frequently occupied sites in single-molecule foot-printing (SMF) data[46] also did not persist upon CTCF degradation (**Supplementary Fig.10b-c**). Given the near-complete ablation of cohesin loops after CTCF degradation (**Fig. 3f-g**), we suggest that the persistent residual CTCF proteins, if any, are insufficient to halt or position cohesin.

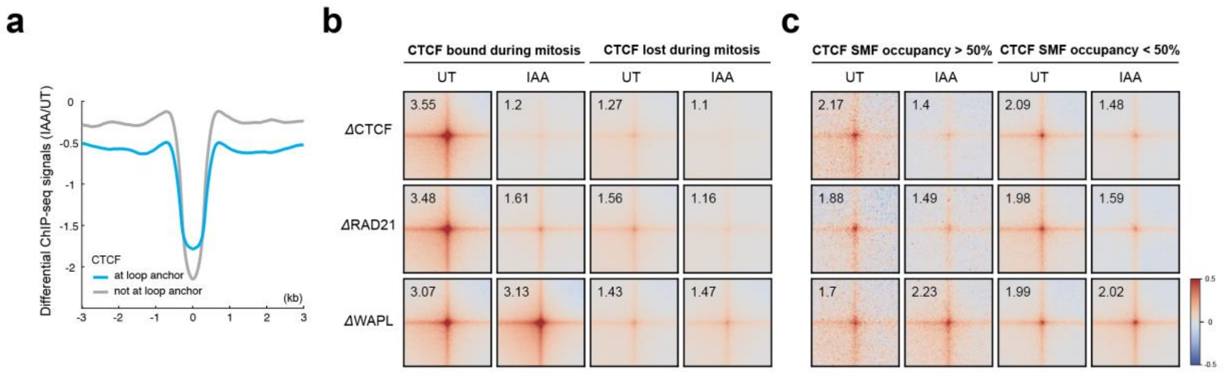

Supplementary Fig. 10 a) Profile of differential ChIP-seq signal for CTCF in a ±3-kb window around loop anchors (gray) or non-loop anchors (blue). b) APA for loops where CTCF is bound (left) or lost (right) at the anchors during mitosis. c) APA for loops with either high (left) or low (right) CTCF occupancy characterized by single-molecular footprinting (SMF) assay at their anchors7.

V. Additional introduction and analysis of YY1

1. Probing YY1 as a candidate regulator of E-P and P-P links

Our finding that E-P and P-P interactions remain largely intact in the absence CTCF, cohesin, and WAPL suggests that other proteins are likely responsible for E-P and P-P interactions. To address this, we searched for factors specifically enriched at E-P and P-P loop anchors. BRD2, BRD4, P300, ESRRB, SP1, Mediator, YY1, pluripotency TFs, and chromatin remodelers are all broadly enriched at enhancer and promoter loop anchors (**Supplementary Fig. 7**). Among them, Mediator has been proposed to function as a structural complex to bridge E-P interactions[47] and promote the folding of sub-TAD structures[48], but two recent studies showed that loss of Mediator does not strongly affect E-P interactions[49,50].

We therefore instead focused on YY1, a multifunctional zinc finger-containing TF (**Extended Data Fig. 7a**) that is ubiquitously expressed, highly conserved, and essential for embryonic development in mammals[51]. Heterozygous YY1 mutations cause Gabriele-de Vries syndrome, which is characterized by developmental delay and intellectual disability[52]. YY1 has been implicated in chromatin looping, particularly during early neural lineage commitment[53], and a previous study suggested it to be a master structural regulator of E-P interactions[54]. However, YY1 redistribution on chromatin triggers little or no change in H3K27ac and H3K27ac-anchored HiChIP interactions upon rapid induction of erythroid differentiation[55]. These confounding results led us to investigate the role, if any, of YY1 in mediating E-P interactions using Micro-C and YY1 depletion.

2. Spatial cluster analysis of YY1

To further link the spatial relationship of YY1 proteins with their different diffusion rates, we reconstructed the spatial distributions of trajectories by their likelihood of diffusion coefficients at 0.02, 0.1, 1, 5, and 20 $\mu m^2 s^{-1}$. Consistent with the live-cell imaging and PALM results, the immobile fraction showed apparent cluster-like structures with ~12 – 30 trajectories per spot (from a total of ~15,000 trajectories per cell), while faster moving YY1 subpopulations showed weaker propensity to cluster within a constrained area (**Supplementary Fig. 11a**). Overlays of the immobile trajectories with the other fractions showed that the molecules within the bound regime had nearly complete overlap, but the faster-diffusing molecules were less likely to co-localize with the immobile clusters (**Supplementary Fig. 11b**). The averaged (**Supplementary Fig. 11b**, insets) and differential cluster signals (**Supplementary Fig. 11b**, bottom panel) between the immobile and the other fractions (**Supplementary Fig. 11a**) further confirmed that the faster-moving molecules more frequently travel to areas in the vicinity of the immobile clusters. These results are consistent with chromatin bound YY1 proteins forming clusters, while the diffusing YY1 molecules appear to search the 3D nuclear space outside of the clusters. We previously made similar observations for CTCF[56–58].

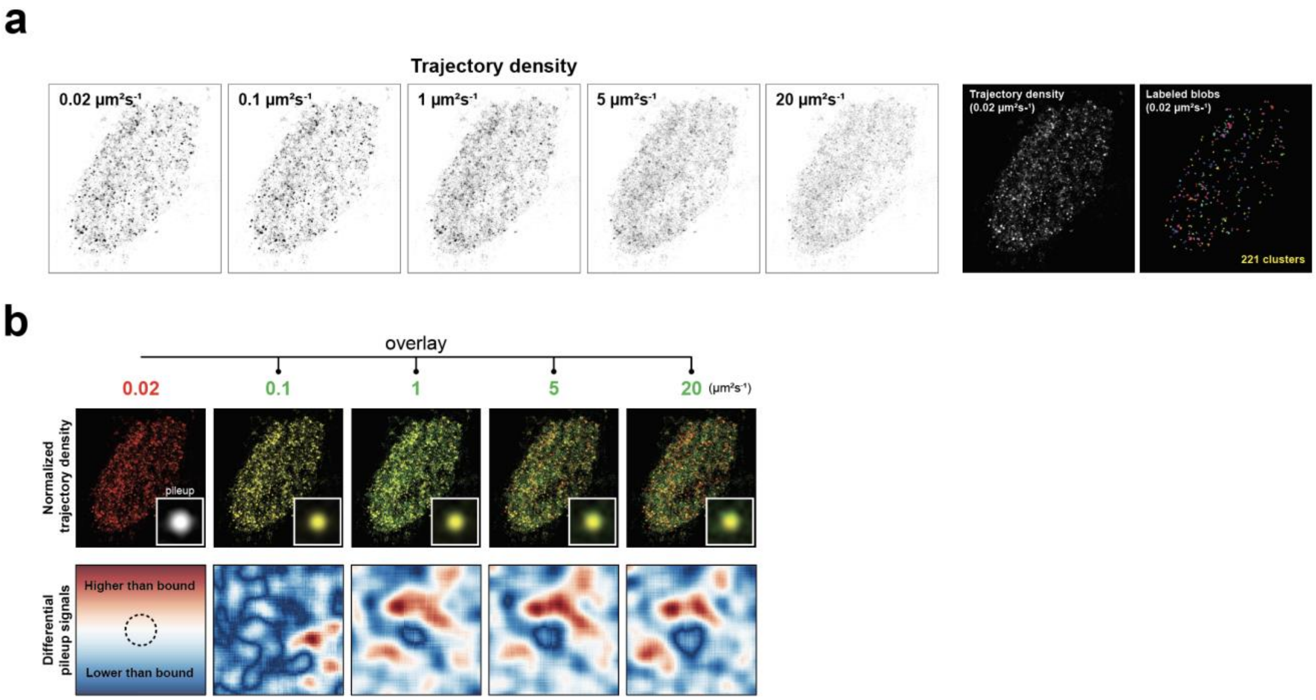

Supplementary Fig. 11. a) Spatial reconstruction of spaSPT data for YY1's trajectory densities. YY1 trajectories are binned by diffusion coefficients, as indicated (left). Segmentation of YY1 clusters with the reconstructed images from the spaSPT data. Individual YY1 clusters are colored (right). b) Spatial reconstruction of spaSPT data for YY1's trajectory densities. YY1 trajectories are binned by diffusion coefficients as indicated. The bound trajectories are colored in red. The diffusing trajectories are colored in green and overlaid with the bound trajectories. The insets show the averaged signals for all identified clusters aggregating at the center of the plot with the same imaging overlays. Differential signals comparing each

3. Dynamics of YY1 vs CTCF

While YY1's average residence time of ~13-60 seconds is similar to that of many TFs, it is much shorter than the residence times of known structural factors such as CTCF (~1-4 min) and cohesin in G1 (~20-25 min) in mESCs[56]. These results may explain why YY1 depletion has a marginal effect on chromatin looping compared to CTCF and cohesin depletion. CTCF/cohesin loops are generally stronger and almost completely lost upon CTCF/cohesin depletion (**Fig. 3f-g**), whereas YY1 loops tend to be weaker and less affected by YY1 depletion (**Fig. 5e&g**). Furthermore, the chromatin bound fraction of YY1 (~30%) is considerably smaller than that for CTCF (~50-70%)[56].

VI. Additional discussion

1. Consideration of phase-separation mechanism for gene regulation

Phase-separated transcriptional condensates have been proposed to mediate gene activation and E-P interactions[59]. Pol II, Mediator, BRD4, and many TFs containing intrinsically disordered regions (IDRs) that tend to aggregate into local-high concentration hubs in the nucleus[60–62]. Hub formation is thought to be a general property of TFs used to engage with regulatory elements and keep them in the spatial vicinity for gene regulation[59–62]. While not specifically addressed in this study, recent studies employing acute depletion/inhibition of Mediator and BRD4, which are supposed to be key players in condensate formation, also found no drastic effects on E-P interactions[49,50,63]. Thus, condensates also do not seem to be generally required for the maintenance of E-P interactions.

2. Evidence of CTCF and cohesin regulates gene expression

The evidence that CTCF and cohesin can directly or indirectly regulate E-P interactions and affect gene expression in many cases is overwhelming. This evidence includes the following: 1) Insertion of CTCF sites between an enhancer and a promoter can both reduce E-P interactions and strongly reduce gene expression[64–66]; 2) CTCF binding site silencing[67,68] or genetic CTCF binding site loss[26,69,70] can cause aberrant E-P interactions and gene expression and drive disease; 3) Inversion or repositioning of CTCF sites can redirect E-P interactions that cause gene misexpression and diseases[71,72]. Two recent studies have also proposed variants of a time-buffering model based on mathematical modeling of E-P interactions and gene expression[73,74]. In both models, individual E-P interactions are memorized – either as long-lived promoter states[66] or as long-lived "promoter tags"[73] – such that gene expression can be temporally uncoupled from E-P interactions, yet still be causally linked.

3. Consideration of other mechanisms by which cohesin regulates E-P/P-P loops

We probed the nuclear dynamics of two additional transcription factors, KLF4 and SOX2, by spaSPT and found that their bound fractions were reduced by ~20% 3 hours after cohesin degradation (**Extended Data Fig. 9g**). Furthermore, while our paper was under review an independent study from Gordon Hager's group [75] found precisely the same thing: for the TF

glucocorticoid receptor, cohesin depletion leads to decreased chromatin binding. Thus, while we agree with the reviewer that our model was too strong a leap based on a single TF (YY1) in the original submission, since we now have evidence for 4 TFs (YY1, KLF4, SOX2, GR) in two different cell types (mESCs, human cancer cells) and from two different labs, we now believe that the evidence is strong enough to invoke a role for cohesin in facilitating TF binding.

Finally, the question that "*How is an alternative model in which the act of loop extrusion acts to stir the nucleoplasm, resulting in higher diffusion and faster search times?*". However, loop extrusion has the opposite effect – it makes chromatin diffusion slower. Indeed, two recent studies ([76,77] have both shown that cohesin depletion leads to a ~2-4-fold increase in chromatin diffusion dynamics. However, faster chromatin motion is extremely unlikely to noticeable accelerate the TF target search.

To understand why consider the Smoluchowski equation (Smoluchowski, Versuch einer mathematischen Theorie der Koagulationskinetik kolloider Losungen, 1916) for diffusion-limited bi-molecular rate constants:

$$k_{\mathrm{ON}} = 4\pi(D_{\mathrm{TF}} + D_{\mathrm{DNA}})(R_{\mathrm{TF}} + R_{\mathrm{DNA}})b$$

Where *D* is the diffusion coefficient, *R* is the size of the TF or DNA binding site, and *b* is the cross section. Using typical diffusion coefficients of $D_{\mathrm{TF}} = 3\,\frac{\mu\mathrm{m}^2}{\mathrm{s}}$ and $D_{\mathrm{DNA}} = 0.01\,\frac{\mu\mathrm{m}^2}{\mathrm{s}}$, we can calculate how much TF search would be expected to be speed up by a 4-fold increase in chromatin diffusion (as observed during cohesin depletion):

$$\frac{k_{\mathrm{ON},\Delta\mathrm{RAD21}}}{k_{\mathrm{ON,UT}}} = \frac{4\pi(D_{\mathrm{TF}} + D_{\mathrm{DNA}})(R_{\mathrm{TF}} + R_{\mathrm{DNA}})b}{4\pi(D_{\mathrm{TF}} + D_{\mathrm{DNA}})(R_{\mathrm{TF}} + R_{\mathrm{DNA}})b} = \frac{D_{\mathrm{TF}} + D_{\mathrm{DNA}}}{D_{\mathrm{TF}} + D_{\mathrm{DNA}}} = \frac{3 + 0.04\,\frac{\mu\mathrm{m}^2}{\mathrm{s}}}{3 + 0.01\,\frac{\mu\mathrm{m}^2}{\mathrm{s}}} = 1.01$$

In other words, the TF search time would be expected to increase by 1% due to cohesin depletion based on chemical kinetics. Instead, we observe the opposite – the 2.2-fold slower TF search for YY1. Thus, both the magnitude (1% increase vs. 54% decrease) and the direction (chemical kinetic predicts faster search, we observe much slower search) are inconsistent with the model proposed by the reviewer. In conclusion, we feel confident in ruling out changes in chromatin diffusion due to cohesin depletion as a possible explanation for the reduction in TF search kinetics.

References

1. Yaffe, E. & Tanay, A. Probabilistic modeling of Hi-C contact maps eliminates systematic biases to characterize global chromosomal architecture. *Nat Genet* 43, 1059–65 (2011).

2. Imakaev, M. *et al.* Iterative correction of Hi-C data reveals hallmarks of chromosome organization. *Nat Methods* 9, 999–1003 (2012).

3. Rao, S. S. P. *et al.* A 3D Map of the Human Genome at Kilobase Resolution Reveals Principles of Chromatin Looping. *Cell* 159, 1665–1680 (2014).

4. Chereji, R. V., Bryson, T. D. & Henikoff, S. Quantitative MNase-seq accurately maps nucleosome occupancy levels. *Genome Biol* 20, 198 (2019).

5. Schwartz, U. *et al.* Characterizing the nuclease accessibility of DNA in human cells to map higher order structures of chromatin. *Nucleic Acids Res* 47, 1239–1254 (2018).

6. Hsieh, T.-H. S. *et al.* Resolving the 3D Landscape of Transcription-Linked Mammalian Chromatin Folding. *Mol Cell* 78, 539-553.e8 (2020).

7. Krietenstein, N. *et al.* Ultrastructural Details of Mammalian Chromosome Architecture. *Mol Cell* 78, 554-565.e7 (2020).

8. Hua, P. *et al.* Defining genome architecture at base-pair resolution. *Nature* 1–5 (2021) doi:10.1038/s41586-021-03639-4.

9. Bonev, B. *et al.* Multiscale 3D Genome Rewiring during Mouse Neural Development. *Cell* 171, 557-572.e24 (2017).

10. Nora, E. P. *et al.* Targeted Degradation of CTCF Decouples Local Insulation of Chromosome Domains from Genomic Compartmentalization. *Cell* 169, 930-944.e22 (2017).

11. Liu, N. Q. *et al.* WAPL maintains a cohesin loading cycle to preserve cell-type-specific distal gene regulation. *Nat Genet* 53, 100–109 (2021).

12. Luan, J. *et al.* Distinct properties and functions of CTCF revealed by a rapidly inducible degron system. *Cell Reports* 34, 108783 (2021).

13. Gu, B. *et al.* Opposing Effects of Cohesin and Transcription on CTCF Organization Revealed by Super-resolution Imaging. *Mol Cell* 80, 699-711.e7 (2020).

14. Busslinger, G. A. *et al.* Cohesin is positioned in mammalian genomes by transcription, CTCF and Wapl. *Nature* 544, 503–507 (2017).

15. Lengronne, A. *et al.* Cohesin relocation from sites of chromosomal loading to places of convergent transcription. *Nature* 430, 573–578 (2004).

16. Pugacheva, E. M. *et al.* CTCF mediates chromatin looping via N-terminal domain-dependent cohesin retention. *Proc National Acad Sci* 117, 2020–2031 (2020).

17. Kueng, S. *et al.* Wapl Controls the Dynamic Association of Cohesin with Chromatin. *Cell* 127, 955–967 (2006).

18. Rao, S. S. P. *et al.* Cohesin Loss Eliminates All Loop Domains. *Cell* 171, 305-320.e24 (2017).

19. Wutz, G. *et al.* Topologically associating domains and chromatin loops depend on cohesin and are regulated by CTCF, WAPL, and PDS5 proteins. *Embo J* 36, 3573–3599 (2017).

20. Schwarzer, W. *et al.* Two independent modes of chromatin organization revealed by cohesin removal. *Nature* 551, 51–56 (2017).

21. Haarhuis, J. H. I. *et al.* The Cohesin Release Factor WAPL Restricts Chromatin Loop Extension. *Cell* 169, 693-707.e14 (2017).

22. Fudenberg, G. *et al.* Formation of Chromosomal Domains by Loop Extrusion. *Cell Reports* 15, 2038–2049 (2016).

23. Sanborn, A. L. *et al.* Chromatin extrusion explains key features of loop and domain formation in wild-type and engineered genomes. *Proc National Acad Sci* 112, E6456–E6465 (2015).

24. Zheng, H. & Xie, W. The role of 3D genome organization in development and cell differentiation. *Nat Rev Mol Cell Bio* 20, 535–550 (2019).

25. Robson, M. I., Ringel, A. R. & Mundlos, S. Regulatory Landscaping: How Enhancer-Promoter Communication Is Sculpted in 3D. *Mol Cell* 74, 1110–1122 (2019).

26. Anania, C. *et al.* In vivo dissection of a clustered-CTCF domain boundary reveals developmental principles of regulatory insulation. *Biorxiv* 2021.04.14.439779 (2021) doi:10.1101/2021.04.14.439779.

27. Calderon, L. *et al.* Activity-induced gene expression and long-range enhancer-promoter contacts in cohesin-deficient neurons. *Biorxiv* 2021.02.24.432639 (2021) doi:10.1101/2021.02.24.432639.

28. Stik, G. *et al.* CTCF is dispensable for immune cell transdifferentiation but facilitates an acute inflammatory response. *Nat Genet* 52, 655–661 (2020).

29. Gasperini, M. *et al.* A Genome-wide Framework for Mapping Gene Regulation via Cellular Genetic Screens. *Cell* 176, 377-390.e19 (2019).

30. Sun, F. *et al.* Promoter-Enhancer Communication Occurs Primarily within Insulated Neighborhoods. *Mol Cell* 73, 250-263.e5 (2019).

31. Bintu, B. *et al.* Super-resolution chromatin tracing reveals domains and cooperative interactions in single cells. *Science* 362, eaau1783 (2018).

32. Stevens, T. J. *et al.* 3D structures of individual mammalian genomes studied by single-cell Hi-C. *Nature* 544, 59–64 (2017).

33. Cattoni, D. I. *et al.* Single-cell absolute contact probability detection reveals chromosomes are organized by multiple low-frequency yet specific interactions. *Nat Commun* 8, 1753 (2017).

34. Nagano, T. *et al.* Single-cell Hi-C reveals cell-to-cell variability in chromosome structure. *Nature* 502, 59–64 (2013).

35. Giorgetti, L. *et al.* Predictive Polymer Modeling Reveals Coupled Fluctuations in Chromosome Conformation and Transcription. *Cell* 157, 950–963 (2014).

36. Finn, E. H. *et al.* Extensive Heterogeneity and Intrinsic Variation in Spatial Genome Organization. *Cell* 176, 1502-1515.e10 (2019).

37. Mateo, L. J. *et al.* Visualizing DNA folding and RNA in embryos at single-cell resolution. *Nature* 568, 49–54 (2019).

38. Luppino, J. M. *et al.* Cohesin promotes stochastic domain intermingling to ensure proper regulation of boundary-proximal genes. *Nat Genet* 52, 840–848 (2020).

39. Szabo, Q. *et al.* Regulation of single-cell genome organization into TADs and chromatin nanodomains. *Nat Genet* 52, 1151–1157 (2020).

40. Rodríguez-Carballo, E. *et al.* Chromatin topology and the timing of enhancer function at the HoxD locus. *Proc National Acad Sci* 117, 31231–31241 (2020).

41. Chakraborty, S. *et al.* High affinity enhancer-promoter interactions can bypass CTCF/cohesin-mediated insulation and contribute to phenotypic robustness. *Biorxiv* 2021.12.30.474562 (2022) doi:10.1101/2021.12.30.474562.

42. Lu, L. *et al.* Robust Hi-C Maps of Enhancer-Promoter Interactions Reveal the Function of Non-coding Genome in Neural Development and Diseases. *Mol Cell* (2020) doi:10.1016/j.molcel.2020.06.007.

43. Thiecke, M. J. *et al.* Cohesin-Dependent and -Independent Mechanisms Mediate Chromosomal Contacts between Promoters and Enhancers. *Cell Reports* 32, 107929 (2020).

44. Rowley, M. J. & Corces, V. G. Organizational principles of 3D genome architecture. *Nat Rev Genet* 19, 789–800 (2018).

45. Owens, N. *et al.* CTCF confers local nucleosome resiliency after DNA replication and during mitosis. *Elife* 8, e47898 (2019).

46. Sönmezer, C. *et al.* Molecular Co-occupancy Identifies Transcription Factor Binding Cooperativity In Vivo. *Mol Cell* (2020) doi:10.1016/j.molcel.2020.11.015.

47. Kagey, M. H. *et al.* Mediator and cohesin connect gene expression and chromatin architecture. *Nature* 467, 430–435 (2010).

48. Phillips-Cremins, J. E. *et al.* Architectural Protein Subclasses Shape 3D Organization of Genomes during Lineage Commitment. *Cell* 153, 1281–1295 (2013).

49. Khattabi, L. E. *et al.* A Pliable Mediator Acts as a Functional Rather Than an Architectural Bridge between Promoters and Enhancers. *Cell* 178, 1145-1158.e20 (2019).

50. Jaeger, M. G. *et al.* Selective Mediator dependence of cell-type-specifying transcription. *Nat Genet* 1–9 (2020) doi:10.1038/s41588-020-0635-0.

51. Donohoe, M. E. *et al.* Targeted Disruption of Mouse Yin Yang 1 Transcription Factor Results in Peri-Implantation Lethality. *Mol Cell Biol* 19, 7237–7244 (1999).

52. Gabriele, M. *et al.* YY1 Haploinsufficiency Causes an Intellectual Disability Syndrome Featuring Transcriptional and Chromatin Dysfunction. *Am J Hum Genet* 100, 907–925 (2017).

53. Beagan, J. A. *et al.* YY1 and CTCF orchestrate a 3D chromatin looping switch during early neural lineage commitment. *Genome Res* 27, 1139–1152 (2017).

54. Weintraub, A. S. *et al.* YY1 Is a Structural Regulator of Enhancer-Promoter Loops. *Cell* 171, 1573-1588.e28 (2017).

55. Perreault, A. A., Brown, J. D. & Venters, B. J. Erythropoietin Regulates Transcription and YY1 Dynamics in a Pre-established Chromatin Architecture. *Iscience* 23, 101583 (2020).

56. Hansen, A. S., Pustova, I., Cattoglio, C., Tjian, R. & Darzacq, X. CTCF and cohesin regulate chromatin loop stability with distinct dynamics. *Elife* 6, e25776 (2017).

57. Hansen, A. S. *et al.* Distinct Classes of Chromatin Loops Revealed by Deletion of an RNA-Binding Region in CTCF. *Mol Cell* 76, 395-411.e13 (2019).

58. Hansen, A. S., Amitai, A., Cattoglio, C., Tjian, R. & Darzacq, X. Guided nuclear exploration increases CTCF target search efficiency. *Nat Chem Biol* 16, 257–266 (2020).

59. Hnisz, D., Shrinivas, K., Young, R. A., Chakraborty, A. K. & Sharp, P. A. A Phase Separation Model for Transcriptional Control. *Cell* 169, 13–23 (2017).

60. Sabari, B. R. *et al.* Coactivator condensation at super-enhancers links phase separation and gene control. *Science* 361, eaar3958 (2018).

61. Boija, A. *et al.* Transcription Factors Activate Genes through the Phase-Separation Capacity of Their Activation Domains. *Cell* 175, 1842-1855.e16 (2018).

62. Cho, W.-K. *et al.* Mediator and RNA polymerase II clusters associate in transcription-dependent condensates. *Science* 361, 412–415 (2018).

63. Crump, N. T. *et al.* BET inhibition disrupts transcription but retains enhancer-promoter contact. *Nat Commun* 12, 223 (2021).

64. Hou, C., Zhao, H., Tanimoto, K. & Dean, A. CTCF-dependent enhancer-blocking by alternative chromatin loop formation. *Proc National Acad Sci* 105, 20398–20403 (2008).

65. Huang, H. *et al.* CTCF mediates dosage- and sequence-context-dependent transcriptional insulation by forming local chromatin domains. *Nat Genet* 1–11 (2021) doi:10.1038/s41588-021-00863-6.

66. Zuin, J. *et al.* Nonlinear control of transcription through enhancer-promoter interactions. *Biorxiv* 2021.04.22.440891 (2021) doi:10.1101/2021.04.22.440891.

67. Flavahan, W. A. *et al.* Insulator dysfunction and oncogene activation in IDH mutant gliomas. *Nature* 529, 110–114 (2016).

68. Flavahan, W. A. *et al.* Altered chromosomal topology drives oncogenic programs in SDH-deficient GISTs. *Nature* 575, 229–233 (2019).

69. Lupiáñez, D. G. *et al.* Disruptions of Topological Chromatin Domains Cause Pathogenic Rewiring of Gene-Enhancer Interactions. *Cell* 161, 1012–1025 (2015).

70. Ushiki, A. *et al.* Deletion of CTCF sites in the SHH locus alters enhancer–promoter interactions and leads to acheiropodia. *Nat Commun* 12, 2282 (2021).

71. Despang, A. *et al.* Functional dissection of the Sox9–Kcnj2 locus identifies nonessential and instructive roles of TAD architecture. *Nat Genet* 51, 1263–1271 (2019).

72. Kraft, K. *et al.* Serial genomic inversions induce tissue-specific architectural stripes, gene misexpression and congenital malformations. *Nat Cell Biol* 21, 305–310 (2019).

73. Xiao, J., Hafner, A. & Boettiger, A. N. How subtle changes in 3D structure can create large changes in transcription. *Biorxiv* 2020.10.22.351395 (2020) doi:10.1101/2020.10.22.351395.

74. Zuin, J. *et al.* Nonlinear control of transcription through enhancer–promoter interactions. *Nature* 604, 571–577 (2022).

75. Rinaldi, L. *et al.* The glucocorticoid receptor associates with the cohesin loader NIPBL to promote long-range gene regulation. *Sci Adv* 8, eabj8360 (2022).

76. Gabriele, M. *et al.* Dynamics of CTCF- and cohesin-mediated chromatin looping revealed by live-cell imaging. *Science* 376, 496–501 (2022).

77. Mach, P. *et al.* Live-cell imaging and physical modeling reveal control of chromosome folding dynamics by cohesin and CTCF. *Biorxiv* 2022.03.03.482826 (2022) doi:10.1101/2022.03.03.482826.

78. Filion, G. J. *et al.* Systematic Protein Location Mapping Reveals Five Principal Chromatin Types in Drosophila Cells. *Cell* 143, 212–224 (2010).
