# Supplementary material for: Enhancer–promoter interactions and transcription are largely maintained upon acute loss of CTCF, cohesin, WAPL or YY1
Source: Nat Genet. 2022 Dec 5;54(12):1919–32. doi: 10.1038/s41588-022-01223-8 (PMC9729117; doi:10.1038/s41588-022-01223-8)
Supplement: Supplementary file 2 — Reporting Summary [file 41588_2022_1223_MOESM2_ESM.pdf]

nature portfolio

Corresponding author(s): Robert Tjian

Last updated by author(s): Aug 16, 2022

Reporting Summary

Nature Portfolio wishes to improve the reproducibility of the work that we publish. This form provides structure for consistency and transparency in reporting. For further information on Nature Portfolio policies, see our Editorial Policies and the Editorial Policy Checklist.

Statistics

For all statistical analyses, confirm that the following items are present in the figure legend, table legend, main text, or Methods section.

n/a	Confirmed	
☐	☒	The exact sample size (*n*) for each experimental group/condition, given as a discrete number and unit of measurement
☐	☒	A statement on whether measurements were taken from distinct samples or whether the same sample was measured repeatedly
☐	☒	The statistical test(s) used AND whether they are one- or two-sided<br>*Only common tests should be described solely by name; describe more complex techniques in the Methods section.*
☒	☐	A description of all covariates tested
☐	☒	A description of any assumptions or corrections, such as tests of normality and adjustment for multiple comparisons
☐	☒	A full description of the statistical parameters including central tendency (e.g. means) or other basic estimates (e.g. regression coefficient) AND variation (e.g. standard deviation) or associated estimates of uncertainty (e.g. confidence intervals)
☐	☒	For null hypothesis testing, the test statistic (e.g. *F*, *t*, *r*) with confidence intervals, effect sizes, degrees of freedom and *P* value noted<br>*Give P values as exact values whenever suitable.*
☒	☐	For Bayesian analysis, information on the choice of priors and Markov chain Monte Carlo settings
☒	☐	For hierarchical and complex designs, identification of the appropriate level for tests and full reporting of outcomes
☐	☒	Estimates of effect sizes (e.g. Cohen's *d*, Pearson's *r*), indicating how they were calculated

Our web collection on statistics for biologists contains articles on many of the points above.

Software and code

Policy information about availability of computer code

Data collection	Nikon NIS-Elements software (v 4.6) for collecting single-molecule imaging<br>Zeiss Zen Blue imaging software (v 3.1) for collecting confocal and Airyscan imaging
Data analysis	Sequencing analysis: TrimGalore (v 0.6.5) (https://github.com/FelixKrueger/TrimGalore), Bowtie2 (v 2.3.0) (http://bowtie-bio.sourceforge.net/bowtie2/index.shtml), samtools (v 1.9) (http://www.htslib.org/), deepTools (v 2.4.1 & v 3.5.0) (https://github.com/deeptools/deepTools), MACS (v 2.2.6) (https://github.com/macs3-project/MACS), MAnorm2 (v 1.0) (https://github.com/tushiqi/MAnorm2), HiC-Pro (v 2.11.3) (https://github.com/nservant/HiC-Pro), COOLER (v 0.8.10) (https://github.com/open2c/cooler), JUICER (v 1.22.01) (https://github.com/aidenlab/juicer), HiGlass (v 1.11.7) (https://github.com/higlass/higlass), cooltools (v 0.3.2) (https://github.com/open2c/cooltools), 3DChromatin_ReplicateQC (v 1.0.1) (https://github.com/kundajelab/3DChromatin_ReplicateQC), coolpuppy (v 0.9.5) (https://github.com/open2c/coolpuppy), Mustache (v 1.0.1) (https://github.com/ay-lab/mustache), chromosight (v 0.9.8) (https://github.com/koszullab/chromosight), chromHMM (v 1.22) (http://compbio.mit.edu/ChromHMM/), Bedtools (v 2.30.0) (https://bedtools.readthedocs.io/en/latest/index.html), kallisto (0.46.2) (https://github.com/pachterlab/kallisto), DeSeq2 (v 1.30.1) (https://bioconductor.org/packages/release/bioc/html/DESeq2.html), NRSA (v 2.0) (http://bioinfo.vanderbilt.edu/NRSA/), Rsubread (v 1.22.2) (https://bioconductor.org/packages/release/bioc/html/Rsubread.html)<br>Imaging analysis: ImageJ (v 1.53c) (https://imagej.nih.gov/ij/), Spot-on (v 1.0.4) (https://gitlab.com/tjian-darzacq-lab/spot-on-matlab), quot (v 3.0) (https://github.com/alecheckert/quot), SASPT (v 1.0) (https://github.com/alecheckert/saspt), Thunderstorm (v 1.3) (https://github.com/zitmen/thunderstorm)<br>Flow cytomotry analysis: FlowJo (v 10.3) (https://www.flowjo.com/)

For manuscripts utilizing custom algorithms or software that are central to the research but not yet described in published literature, software must be made available to editors and reviewers. We strongly encourage code deposition in a community repository (e.g. GitHub). See the Nature Portfolio guidelines for submitting code & software for further information.

Data

Policy information about availability of data

All manuscripts must include a data availability statement. This statement should provide the following information, where applicable:

- Accession codes, unique identifiers, or web links for publicly available datasets
- A description of any restrictions on data availability
- For clinical datasets or third party data, please ensure that the statement adheres to our policy

The Micro-C, ChIP-seq, nascent RNA-seq and total RNA-seq data generated in this publication are available in NCBI Gene Expression Omnibus through GEO Series accession number GSE178982. We also reanalyzed data that we previously generated in wild type mESCs (GSE130275). spaSPT raw data are accessible through DOI: 10.5281/zenodo.5035837. The reference genome mm10 and sacCer3 are available through UCSC genome browser (https://hgdownload.soe.ucsc.edu/downloads.html).

Human research participants

Policy information about studies involving human research participants and Sex and Gender in Research.

Reporting on sex and gender	n/a
Population characteristics	n/a
Recruitment	n/a
Ethics oversight	n/a

Note that full information on the approval of the study protocol must also be provided in the manuscript.

Field-specific reporting

Please select the one below that is the best fit for your research. If you are not sure, read the appropriate sections before making your selection.

☒ Life sciences ☐ Behavioural & social sciences ☐ Ecological, evolutionary & environmental sciences

For a reference copy of the document with all sections, see nature.com/documents/nr-reporting-summary-flat.pdf

Life sciences study design

All studies must disclose on these points even when the disclosure is negative.

Sample size	1) Confocal/Airyscan imaging is mainly qualitative. We routinely collected at least six regions of interest (ROIs) with two different cell cultures on two separate days; 2) FRAP: We generally collected data from at least 6 cells per cell line per condition per day, and all presented data are from at least two independent replicates on different days; 3) SPT: We recorded movies for six cells per cell line or condition per day, and all data presented are from at least two independent experiments conducted corresponding to at least 12 cells and at least 100,000 localizations. No statistical method was used to predetermine sample size. We chose the sample size as suggested in the previous report (doi: 10.7554/elife.25776).
Data exclusions	1) FRAP: We excluded data if the bleached spot is not detectable by our algorithm, and if the cell drifted away from the focus during acquisition; 2) SPT: We excluded data if the total localization was lower than 20,000 per cell.
Replication	Sequencing data: We generally collected at least 2 biological replicates per condition per day to gain statistical power. For some samples (Micro-C_ΔCTCF_IAA, Micro-C_ΔRAD21_IAA, Micro-C_ΔWAPL_IAA), we performed pilot tests so that the sample sizes will increase to 4.<br>Imaging data: We generally collected at least 2 biological replicates per condition per day.<br>Immunoblotting, biochemical fractionation, flow cytometry experiments were repeated and confirmed at least twice.<br>All attempts at replication were successful.
Randomization	Samples were divided into groups based on genomic perturbations. Each auxin-degradation sample is coupled with untreated control.
Blinding	The study does not involve therapeutic or animal experiments, so blinding was not necessary. The labeling of samples is also required for all computational analyses.

Reporting for specific materials, systems and methods

We require information from authors about some types of materials, experimental systems and methods used in many studies. Here, indicate whether each material, system or method listed is relevant to your study. If you are not sure if a list item applies to your research, read the appropriate section before selecting a response.

Materials & experimental systems

n/a	Involved in the study
☐	☒ Antibodies
☐	☒ Eukaryotic cell lines
☒	☐ Palaeontology and archaeology
☒	☐ Animals and other organisms
☒	☐ Clinical data
☒	☐ Dual use research of concern

Methods

n/a	Involved in the study
☐	☒ ChIP-seq
☐	☒ Flow cytometry
☒	☐ MRI-based neuroimaging

Antibodies

Antibodies used

anti-CTCF Novus NBP2-52909
anti-CTCF EMD 07-729
anti-CTCF Abcam ab128873
anti-Halo Promega G921A
anti-RAD21 EMD 05-908
anti-RAD21 Abcam ab154769
anti-RAD21 Abcam ab154769
anti-V5 ThermoFisher R960-25
anti-YY1 Santa Cruz Biotechnology sc-7341
anti-YY1 Abcam ab38422
anti-YY1 Abcam ab109237
anti-YY1 Bethyl Laboratories Inc. A302-778A
anti-YY1 Bethyl Laboratories Inc. A302-779A
anti-RFP / anti-mScarletI Chromotek 6G6
anti-RFP / anti-mScarletI Rockland 600-401-379
anti-WAPL Proteintech 16370-1-AP
anti-HA Abcam ab9110
anti-ACTB Sigma A2228
anti-OCT4 Santa Cruz Biotechnology sc-8628
anti-TBP abcam ab51841
anti-H2B ThermoFisher MA524697
anti-SMC1A Bethyl laboratories A300-055A
anti-SMC3 Bethyl laboratories A300-060A

Validation

CTCF, RAD21, WAPL, YY1, HaloTag, V5, HA, RFP antibodies were validated by WB in cells depleted with the corresponding protein. SMC1A and SMC3 were validated by ChIP-seq, which signals are largely overlapped with RAD21. ACTB, TBP, OCT4, and H2B antibodies are well-validated by various studies and manufacturers.

Eukaryotic cell lines

Policy information about cell lines and Sex and Gender in Research

Cell line source(s)

JM8.N4 mESC was obtained from the KOMP Repository at UC Davis.

Authentication

JM8.N4 mESCs were authenticated by whole-genome sequencing and morphology.

Mycoplasma contamination

JM8.N4 mESCs were pathogen tested using the IMPACT II test by IDEXX BioResearch (Westbrook, ME). All cells were negative for all pathogens, including Ectromelia, EDIM, LCMV, LDEV, MAV1, MAV2, mCMV, MHV, MNV, MPV, MVM, Mycoplasma pulmonis, Mycoplasma sp., Polyoma, PVM, REO3, Sendai, and TMEV.

Commonly misidentified lines
(See ICLAC register)

No commonly misidentified line was used.

ChIP-seq

Data deposition

☒ Confirm that both raw and final processed data have been deposited in a public database such as GEO.

☒ Confirm that you have deposited or provided access to graph files (e.g. BED files) for the called peaks.

Data access links
May remain private before publication.

https://www.ncbi.nlm.nih.gov/geo/query/acc.cgi?acc=GSE178982

Files in database submission

RTCC51A_S1_L001_R1_001.fastq.bz2
RTCC51B_S2_L001_R1_001.fastq.bz2
RTCC51C_S3_L001_R1_001.fastq.bz2
RTCC51D_S4_L001_R1_001.fastq.bz2
RTCC51E_S5_L001_R1_001.fastq.bz2
RTCC51F_S6_L001_R1_001.fastq.bz2
RTCC51G_S7_L001_R1_001.fastq.bz2
RTCC51H_S8_L001_R1_001.fastq.bz2
RTCC51I_S9_L001_R1_001.fastq.bz2
RTCC51J_S10_L001_R1_001.fastq.bz2
RTCC51K_S11_L001_R1_001.fastq.bz2
RTCC51L_S12_L001_R1_001.fastq.bz2
RTCC52A_S13_L002_R1_001.fastq.bz2
RTCC52B_S14_L002_R1_001.fastq.bz2
RTCC52C_S15_L002_R1_001.fastq.bz2
RTCC52D_S16_L002_R1_001.fastq.bz2
RTCC52E_S17_L002_R1_001.fastq.bz2
RTCC52F_S18_L002_R1_001.fastq.bz2
RTCC52G_S19_L002_R1_001.fastq.bz2
RTCC52H_S20_L002_R1_001.fastq.bz2
RTCC52I_S21_L002_R1_001.fastq.bz2
RTCC52J_S22_L002_R1_001.fastq.bz2
RTCC52K_S23_L002_R1_001.fastq.bz2
RTCC52L_S24_L002_R1_001.fastq.bz2
RTCC53A_S25_L003_R1_001.fastq.bz2
RTCC53B_S26_L003_R1_001.fastq.bz2
RTCC53C_S27_L003_R1_001.fastq.bz2
RTCC53D_S28_L003_R1_001.fastq.bz2
RTCC53E_S29_L003_R1_001.fastq.bz2
RTCC53F_S30_L003_R1_001.fastq.bz2
RTCC53G_S31_L003_R1_001.fastq.bz2
RTCC53H_S32_L003_R1_001.fastq.bz2
RTCC53I_S33_L003_R1_001.fastq.bz2
RTCC53J_S34_L003_R1_001.fastq.bz2
RTCC53K_S35_L003_R1_001.fastq.bz2
RTCC53L_S36_L003_R1_001.fastq.bz2
RTCC54A_S37_L004_R1_001.fastq.bz2
RTCC54B_S38_L004_R1_001.fastq.bz2
RTCC54C_S39_L004_R1_001.fastq.bz2
RTCC54D_S40_L004_R1_001.fastq.bz2
RTCC54E_S41_L004_R1_001.fastq.bz2
RTCC54F_S42_L004_R1_001.fastq.bz2
RTCC54G_S43_L004_R1_001.fastq.bz2
RTCC54H_S44_L004_R1_001.fastq.bz2
RTCC54I_S45_L004_R1_001.fastq.bz2
RTCC54J_S46_L004_R1_001.fastq.bz2
RTCC54K_S47_L004_R1_001.fastq.bz2
RTCC54L_S48_L004_R1_001.fastq.bz2
RTCC55A_S49_L005_R1_001.fastq.bz2
RTCC55B_S50_L005_R1_001.fastq.bz2
RTCC55C_S51_L005_R1_001.fastq.bz2
RTCC55D_S52_L005_R1_001.fastq.bz2
RTCC55E_S53_L005_R1_001.fastq.bz2
RTCC55F_S54_L005_R1_001.fastq.bz2
RTCC55G_S55_L005_R1_001.fastq.bz2
RTCC55H_S56_L005_R1_001.fastq.bz2
RTCC55I_S57_L005_R1_001.fastq.bz2
RTCC55J_S58_L005_R1_001.fastq.bz2
RTCC55K_S59_L005_R1_001.fastq.bz2
RTCC55L_S60_L005_R1_001.fastq.bz2
RTCC56A_S61_L006_R1_001.fastq.bz2
RTCC56B_S62_L006_R1_001.fastq.bz2
RTCC56C_S63_L006_R1_001.fastq.bz2
RTCC56D_S64_L006_R1_001.fastq.bz2
RTCC56E_S65_L006_R1_001.fastq.bz2
RTCC56F_S66_L006_R1_001.fastq.bz2
RTCC56G_S67_L006_R1_001.fastq.bz2
RTCC56H_S68_L006_R1_001.fastq.bz2
RTCC56I_S69_L006_R1_001.fastq.bz2
RTCC56J_S70_L006_R1_001.fastq.bz2
RTCC56K_S71_L006_R1_001.fastq.bz2
RTCC56L_S72_L006_R1_001.fastq.bz2
RTCC57A_S73_L007_R1_001.fastq.bz2
RTCC57B_S74_L007_R1_001.fastq.bz2
RTCC57C_S75_L007_R1_001.fastq.bz2

RTCC57D_S76_L007_R1_001.fastq.bz2
RTCC57E_S77_L007_R1_001.fastq.bz2
RTCC57F_S78_L007_R1_001.fastq.bz2
RTCC57G_S79_L007_R1_001.fastq.bz2
RTCC57H_S80_L007_R1_001.fastq.bz2
RTCC57I_S81_L007_R1_001.fastq.bz2
RTCC57J_S82_L007_R1_001.fastq.bz2
RTCC57K_S83_L007_R1_001.fastq.bz2
RTCC57L_S84_L007_R1_001.fastq.bz2
RTCC58A_S85_L008_R1_001.fastq.bz2
RTCC58B_S86_L008_R1_001.fastq.bz2
RTCC58C_S87_L008_R1_001.fastq.bz2
RTCC58D_S88_L008_R1_001.fastq.bz2
RTCC58E_S89_L008_R1_001.fastq.bz2
RTCC58F_S90_L008_R1_001.fastq.bz2
RTCC58G_S91_L008_R1_001.fastq.bz2
RTCC58H_S92_L008_R1_001.fastq.bz2
RTCC58I_S93_L008_R1_001.fastq.bz2
RTCC58J_S94_L008_R1_001.fastq.bz2
RTCC58K_S95_L008_R1_001.fastq.bz2
RTCC58L_S96_L008_R1_001.fastq.bz2
RTCC66A_S39_L004_R1_001.fastq.bz2
RTCC66E_S43_L004_R1_001.fastq.bz2
RTCC66B_S40_L004_R1_001.fastq.bz2
RTCC66F_S44_L004_R1_001.fastq.bz2
RTCC67A_S49_L005_R1_001.fastq.bz2
RTCC67B_S50_L005_R1_001.fastq.bz2
RTCC66C_S41_L004_R1_001.fastq.bz2
RTCC66G_S45_L004_R1_001.fastq.bz2
RTCC66D_S42_L004_R1_001.fastq.bz2
RTCC66H_S46_L004_R1_001.fastq.bz2
RTCC67C_S51_L005_R1_001.fastq.bz2
RTCC67D_S52_L005_R1_001.fastq.bz2
CTCF_degron_ut_input_merged_coverage.bw
CTCF_degron_ut_CTCF_IP_merged_coverage.bw
CTCF_degron_ut_RAD21_IP_merged_coverage.bw
CTCF_degron_ut_SMC1A_IP_merged_coverage.bw
CTCF_degron_ut_SMC3_IP_merged_coverage.bw
CTCF_degron_ut_YY1_IP_merged_coverage.bw
CTCF_degron_auxin_input_merged_coverage.bw
CTCF_degron_auxin_CTCF_IP_merged_coverage.bw
CTCF_degron_auxin_RAD21_IP_merged_coverage.bw
CTCF_degron_auxin_SMC1A_IP_merged_coverage.bw
CTCF_degron_auxin_SMC3_IP_merged_coverage.bw
CTCF_degron_auxin_YY1_IP_merged_coverage.bw
RAD21_degron_ut_input_merged_coverage.bw
RAD21_degron_ut_CTCF_IP_merged_coverage.bw
RAD21_degron_ut_RAD21_IP_merged_coverage.bw
RAD21_degron_ut_SMC1A_IP_merged_coverage.bw
RAD21_degron_ut_SMC3_IP_merged_coverage.bw
RAD21_degron_ut_YY1_IP_merged_coverage.bw
RAD21_degron_auxin_input_merged_coverage.bw
RAD21_degron_auxin_CTCF_IP_merged_coverage.bw
RAD21_degron_auxin_RAD21_IP_merged_coverage.bw
RAD21_degron_auxin_SMC1A_IP_merged_coverage.bw
RAD21_degron_auxin_SMC3_IP_merged_coverage.bw
RAD21_degron_auxin_YY1_IP_merged_coverage.bw
WAPL_degron_ut_input_merged_coverage.bw
WAPL_degron_ut_CTCF_IP_merged_coverage.bw
WAPL_degron_ut_RAD21_IP_merged_coverage.bw
WAPL_degron_ut_SMC1A_IP_merged_coverage.bw
WAPL_degron_ut_SMC3_IP_merged_coverage.bw
WAPL_degron_ut_YY1_IP_merged_coverage.bw
WAPL_degron_auxin_input_merged_coverage.bw
WAPL_degron_auxin_CTCF_IP_merged_coverage.bw
WAPL_degron_auxin_RAD21_IP_merged_coverage.bw
WAPL_degron_auxin_SMC1A_IP_merged_coverage.bw
WAPL_degron_auxin_SMC3_IP_merged_coverage.bw
WAPL_degron_auxin_YY1_IP_merged_coverage.bw
YY1_degron_ut_input_merged_coverage.bw
YY1_degron_ut_CTCF_IP_merged_coverage.bw
YY1_degron_ut_RAD21_IP_merged_coverage.bw
YY1_degron_ut_SMC1A_IP_merged_coverage.bw
YY1_degron_ut_SMC3_IP_merged_coverage.bw
YY1_degron_ut_YY1_IP_merged_coverage.bw
YY1_degron_auxin_input_merged_coverage.bw

YY1_degron_auxin_CTCF_IP_merged_coverage.bw
YY1_degron_auxin_RAD21_IP_merged_coverage.bw
YY1_degron_auxin_SMC1A_IP_merged_coverage.bw
YY1_degron_auxin_SMC3_IP_merged_coverage.bw
YY1_degron_auxin_YY1_IP_merged_coverage.bw
YY1_degron_ut_input_rep8_coverage_spikeIn.bw
YY1_degron_ut_input_rep9_coverage_spikeIn.bw
YY1_degron_ut_RFP_IP_rep8_coverage_spikeIn.bw
YY1_degron_ut_RFP_rep9_coverage_spikeIn.bw
YY1_degron_ut_YY1_abcam_IP_coverage_spikeIn.bw
YY1_degron_ut_YY1_bethyl_IP_coverage_spikeIn.bw
YY1_degron_auxin_input_rep8_coverage_spikeIn.bw
YY1_degron_auxin_input_rep9_coverage_spikeIn.bw
YY1_degron_auxin_RFP_IP_rep8_coverage_spikeIn.bw
YY1_degron_auxin_RFP_rep9_coverage_spikeIn.bw
YY1_degron_auxin_YY1_abcam_IP_coverage_spikeIn.bw
YY1_degron_auxin_YY1_bethyl_IP_coverage_spikeIn.bw

Genome browser session
(e.g. UCSC)

no longer applicable

Methodology

Replicates

We performed Spearman's correlation and Jaccard index to assess the reproducibility between samples. Please see Extended Figure 3a-d for the full result and description.

Sequencing depth

Read length: 51 bp
Single-end
File name Replicate Total reads Uniquely mapped reads
RTCC51A_S1_L001_R1_001.fastq.bz2 DCTCF_UT_CTCF_input_Rep1 28390977 21231461
RTCC51B_S2_L001_R1_001.fastq.bz2 DCTCF_UT_CTCF_ChIP_Rep1 32931783 27107390
RTCC51C_S3_L001_R1_001.fastq.bz2 DCTCF_UT_RAD21_ChIP_Rep1 29663143 22152005
RTCC51D_S4_L001_R1_001.fastq.bz2 DCTCF_UT_SMC1A_ChIP_Rep1 31920364 23816575
RTCC51E_S5_L001_R1_001.fastq.bz2 DCTCF_UT_SMC3_ChIP_Rep1 33634052 25358448
RTCC51F_S6_L001_R1_001.fastq.bz2 DCTCF_UT_YY1_ChIP_Rep1 33959459 24136167
RTCC51G_S7_L001_R1_001.fastq.bz2 DCTCF_IAA_YY1_ChIP_Rep1 35069865 26193557
RTCC51H_S8_L001_R1_001.fastq.bz2 DCTCF_IAA_CTCF_input_Rep1 31335397 23944570
RTCC51I_S9_L001_R1_001.fastq.bz2 DCTCF_IAA_CTCF_ChIP_Rep1 29485544 21870596
RTCC51J_S10_L001_R1_001.fastq.bz2 DCTCF_IAA_RAD21_ChIP_Rep1 36571058 26903685
RTCC51K_S11_L001_R1_001.fastq.bz2 DCTCF_IAA_SMC1A_ChIP_Rep1 34239220 25580983
RTCC51L_S12_L001_R1_001.fastq.bz2 DCTCF_IAA_SMC3_ChIP_Rep1 34809582 24676494
RTCC52A_S13_L002_R1_001.fastq.bz2 DRAD21_UT_CTCF_input_Rep1 25144424 18553371
RTCC52B_S14_L002_R1_001.fastq.bz2 DRAD21_UT_CTCF_ChIP_Rep1 35955736 29096124
RTCC52C_S15_L002_R1_001.fastq.bz2 DRAD21_UT_RAD21_ChIP_Rep1 27333608 20517789
RTCC52D_S16_L002_R1_001.fastq.bz2 DRAD21_UT_SMC1A_ChIP_Rep1 29481356 21800492
RTCC52E_S17_L002_R1_001.fastq.bz2 DRAD21_UT_SMC3_ChIP_Rep1 29484547 22039721
RTCC52F_S18_L002_R1_001.fastq.bz2 DRAD21_UT_YY1_ChIP_Rep1 36126503 25657564
RTCC52G_S19_L002_R1_001.fastq.bz2 DRAD21_IAA_YY1_ChIP_Rep1 30035710 22320231
RTCC52H_S20_L002_R1_001.fastq.bz2 DRAD21_IAA_CTCF_input_Rep1 29583069 23915783
RTCC52I_S21_L002_R1_001.fastq.bz2 DRAD21_IAA_CTCF_ChIP_Rep1 40806074 30018724
RTCC52J_S22_L002_R1_001.fastq.bz2 DRAD21_IAA_RAD21_ChIP_Rep1 37048981 27159280
RTCC52K_S23_L002_R1_001.fastq.bz2 DRAD21_IAA_SMC1A_ChIP_Rep1 32725809 24278048
RTCC52L_S24_L002_R1_001.fastq.bz2 DRAD21_IAA_SMC3_ChIP_Rep1 34037754 23713201
RTCC53A_S25_L003_R1_001.fastq.bz2 DWAPL_UT_CTCF_input_Rep1 24384191 18237846
RTCC53B_S26_L003_R1_001.fastq.bz2 DWAPL_UT_CTCF_ChIP_Rep1 33538857 27889963
RTCC53C_S27_L003_R1_001.fastq.bz2 DWAPL_UT_RAD21_ChIP_Rep1 25117348 18995694
RTCC53D_S28_L003_R1_001.fastq.bz2 DWAPL_UT_SMC1A_ChIP_Rep1 32636618 24552472
RTCC53E_S29_L003_R1_001.fastq.bz2 DWAPL_UT_SMC3_ChIP_Rep1 31072690 23659750
RTCC53F_S30_L003_R1_001.fastq.bz2 DWAPL_UT_YY1_ChIP_Rep1 33231275 23748753
RTCC53G_S31_L003_R1_001.fastq.bz2 DWAPL_IAA_YY1_ChIP_Rep1 29212730 21926504
RTCC53H_S32_L003_R1_001.fastq.bz2 DWAPL_IAA_CTCF_input_Rep1 27408935 22403634
RTCC53I_S33_L003_R1_001.fastq.bz2 DWAPL_IAA_CTCF_ChIP_Rep1 24545163 18722085
RTCC53J_S34_L003_R1_001.fastq.bz2 DWAPL_IAA_RAD21_ChIP_Rep1 39372702 29730206
RTCC53K_S35_L003_R1_001.fastq.bz2 DWAPL_IAA_SMC1A_ChIP_Rep1 33064459 25125531
RTCC53L_S36_L003_R1_001.fastq.bz2 DWAPL_IAA_SMC3_ChIP_Rep1 31227728 22462804
RTCC54A_S37_L004_R1_001.fastq.bz2 DYY1_UT_CTCF_input_Rep1 26011907 20213101
RTCC54B_S38_L004_R1_001.fastq.bz2 DYY1_UT_CTCF_ChIP_Rep1 33422785 27745568
RTCC54C_S39_L004_R1_001.fastq.bz2 DYY1_UT_RAD21_ChIP_Rep1 30087634 23277925
RTCC54D_S40_L004_R1_001.fastq.bz2 DYY1_UT_SMC1A_ChIP_Rep1 31801107 24652683
RTCC54E_S41_L004_R1_001.fastq.bz2 DYY1_UT_SMC3_ChIP_Rep1 38307498 30011393
RTCC54F_S42_L004_R1_001.fastq.bz2 DYY1_UT_YY1_ChIP_Rep1 33404639 23732272
RTCC54G_S43_L004_R1_001.fastq.bz2 DYY1_IAA_YY1_ChIP_Rep1 32150295 25007522
RTCC54H_S44_L004_R1_001.fastq.bz2 DYY1_IAA_CTCF_input_Rep1 30418758 25300258
RTCC54I_S45_L004_R1_001.fastq.bz2 DYY1_IAA_CTCF_ChIP_Rep1 29006914 22613689
RTCC54J_S46_L004_R1_001.fastq.bz2 DYY1_IAA_RAD21_ChIP_Rep1 36127905 28137822
RTCC54K_S47_L004_R1_001.fastq.bz2 DYY1_IAA_SMC1A_ChIP_Rep1 34775334 27165767

RTCC54L_S48_L004_R1_001.fastq.bz2 DYY1_IAA_SMC3_ChIP_Rep1 30250760 22575789
RTCC55A_S49_L005_R1_001.fastq.bz2 DCTCF_UT_CTCF_input_Rep2 29064053 21308974
RTCC55B_S50_L005_R1_001.fastq.bz2 DCTCF_UT_CTCF_ChIP_Rep2 28622933 23414213
RTCC55C_S51_L005_R1_001.fastq.bz2 DCTCF_UT_RAD21_ChIP_Rep2 28352119 20707341
RTCC55D_S52_L005_R1_001.fastq.bz2 DCTCF_UT_SMC1A_ChIP_Rep2 31567380 22900366
RTCC55E_S53_L005_R1_001.fastq.bz2 DCTCF_UT_SMC3_ChIP_Rep2 30499910 22361169
RTCC55F_S54_L005_R1_001.fastq.bz2 DCTCF_UT_YY1_ChIP_Rep2 32034114 22436868
RTCC55G_S55_L005_R1_001.fastq.bz2 DCTCF_IAA_YY1_ChIP_Rep2 30759222 23115704
RTCC55H_S56_L005_R1_001.fastq.bz2 DCTCF_IAA_CTCF_input_Rep2 29970750 22561877
RTCC55I_S57_L005_R1_001.fastq.bz2 DCTCF_IAA_CTCF_ChIP_Rep2 26922404 20134454
RTCC55J_S58_L005_R1_001.fastq.bz2 DCTCF_IAA_RAD21_ChIP_Rep2 30922446 22614630
RTCC55K_S59_L005_R1_001.fastq.bz2 DCTCF_IAA_SMC1A_ChIP_Rep2 36670078 27396409
RTCC55L_S60_L005_R1_001.fastq.bz2 DCTCF_IAA_SMC3_ChIP_Rep2 28327056 20318060
RTCC56A_S61_L006_R1_001.fastq.bz2 DRAD21_UT_CTCF_input_Rep2 28533417 21316335
RTCC56B_S62_L006_R1_001.fastq.bz2 DRAD21_UT_CTCF_ChIP_Rep2 39864412 32409731
RTCC56C_S63_L006_R1_001.fastq.bz2 DRAD21_UT_RAD21_ChIP_Rep2 24040588 18207715
RTCC56D_S64_L006_R1_001.fastq.bz2 DRAD21_UT_SMC1A_ChIP_Rep2 33326643 24543818
RTCC56E_S65_L006_R1_001.fastq.bz2 DRAD21_UT_SMC3_ChIP_Rep2 31213208 23450850
RTCC56F_S66_L006_R1_001.fastq.bz2 DRAD21_UT_YY1_ChIP_Rep2 31832642 22608147
RTCC56G_S67_L006_R1_001.fastq.bz2 DRAD21_IAA_YY1_ChIP_Rep2 34373025 25342380
RTCC56H_S68_L006_R1_001.fastq.bz2 DRAD21_IAA_CTCF_input_Rep2 29809808 23571235
RTCC56I_S69_L006_R1_001.fastq.bz2 DRAD21_IAA_CTCF_ChIP_Rep2 25932689 18848066
RTCC56J_S70_L006_R1_001.fastq.bz2 DRAD21_IAA_RAD21_ChIP_Rep2 36267390 26475367
RTCC56K_S71_L006_R1_001.fastq.bz2 DRAD21_IAA_SMC1A_ChIP_Rep2 33420982 24340999
RTCC56L_S72_L006_R1_001.fastq.bz2 DRAD21_IAA_SMC3_ChIP_Rep2 29109019 20424453
RTCC57A_S73_L007_R1_001.fastq.bz2 DWAPL_UT_CTCF_input_Rep2 29365053 21832681
RTCC57B_S74_L007_R1_001.fastq.bz2 DWAPL_UT_CTCF_ChIP_Rep2 31443884 25947147
RTCC57C_S75_L007_R1_001.fastq.bz2 DWAPL_UT_RAD21_ChIP_Rep2 27448853 20709397
RTCC57D_S76_L007_R1_001.fastq.bz2 DWAPL_UT_SMC1A_ChIP_Rep2 33625771 24696151
RTCC57E_S77_L007_R1_001.fastq.bz2 DWAPL_UT_SMC3_ChIP_Rep2 33589486 25104295
RTCC57F_S78_L007_R1_001.fastq.bz2 DWAPL_UT_YY1_ChIP_Rep2 33367843 23747764
RTCC57G_S79_L007_R1_001.fastq.bz2 DWAPL_IAA_YY1_ChIP_Rep2 31873279 23709130
RTCC57H_S80_L007_R1_001.fastq.bz2 DWAPL_IAA_CTCF_input_Rep2 29217470 24308581
RTCC57I_S81_L007_R1_001.fastq.bz2 DWAPL_IAA_CTCF_ChIP_Rep2 27092281 20386332
RTCC57J_S82_L007_R1_001.fastq.bz2 DWAPL_IAA_RAD21_ChIP_Rep2 38029125 28068355
RTCC57K_S83_L007_R1_001.fastq.bz2 DWAPL_IAA_SMC1A_ChIP_Rep2 34460598 25880556
RTCC57L_S84_L007_R1_001.fastq.bz2 DWAPL_IAA_SMC3_ChIP_Rep2 35324429 25450721
RTCC58A_S85_L008_R1_001.fastq.bz2 DYY1_UT_CTCF_input_Rep2 29925152 22627993
RTCC58B_S86_L008_R1_001.fastq.bz2 DYY1_UT_CTCF_ChIP_Rep2 33373385 27186327
RTCC58C_S87_L008_R1_001.fastq.bz2 DYY1_UT_RAD21_ChIP_Rep2 27369432 20907281
RTCC58D_S88_L008_R1_001.fastq.bz2 DYY1_UT_SMC1A_ChIP_Rep2 34935069 26316230
RTCC58E_S89_L008_R1_001.fastq.bz2 DYY1_UT_SMC3_ChIP_Rep2 29912875 22696556
RTCC58F_S90_L008_R1_001.fastq.bz2 DYY1_UT_YY1_ChIP_Rep2 37055177 26986523
RTCC58G_S91_L008_R1_001.fastq.bz2 DYY1_IAA_YY1_ChIP_Rep2 34477481 25774376
RTCC58H_S92_L008_R1_001.fastq.bz2 DYY1_IAA_CTCF_input_Rep2 31577762 25685691
RTCC58I_S93_L008_R1_001.fastq.bz2 DYY1_IAA_CTCF_ChIP_Rep2 28500684 21247823
RTCC58J_S94_L008_R1_001.fastq.bz2 DYY1_IAA_RAD21_ChIP_Rep2 38559687 28706476
RTCC58K_S95_L008_R1_001.fastq.bz2 DYY1_IAA_SMC1A_ChIP_Rep2 34072879 24484758
RTCC58L_S96_L008_R1_001.fastq.bz2 DYY1_IAA_SMC3_ChIP_Rep2 29588719 21873748
RTCC66A_S39_L004_R1_001.fastq.bz2 DYY1_UT_YY1_input_Rep3 31927388 21740506
RTCC66E_S43_L004_R1_001.fastq.bz2 DYY1_UT_YY1_input_Rep4 35563739 23903736
RTCC66B_S40_L004_R1_001.fastq.bz2 DYY1_UT_YY1_ChIP_anti-RFP_Rep1 42139234 26749526
RTCC66F_S44_L004_R1_001.fastq.bz2 DYY1_UT_YY1_ChIP_anti-RFP_Rep2 38365398 24266302
RTCC67A_S39_L004_R1_001.fastq.bz2 DYY1_UT_YY1_ChIP_Rep3 23956428 15852867
RTCC67B_S40_L004_R1_001.fastq.bz2 DYY1_UT_YY1_ChIP_Rep4 31129254 20309199
RTCC66C_S41_L004_R1_001.fastq.bz2 DYY1_IAA_YY1_inpIAA_Rep3 29954279 20441546
RTCC66G_S45_L004_R1_001.fastq.bz2 DYY1_IAA_YY1_inpIAA_Rep4 34928439 23263109
RTCC66D_S42_L004_R1_001.fastq.bz2 DYY1_IAA_YY1_ChIP_anti-RFP_Rep1 34351731 22521528
RTCC66H_S46_L004_R1_001.fastq.bz2 DYY1_IAA_YY1_ChIP_anti-RFP_Rep2 32396014 21297719
RTCC67C_S41_L004_R1_001.fastq.bz2 DYY1_IAA_YY1_ChIP_Rep3 25209642 16904397
RTCC67D_S42_L004_R1_001.fastq.bz2 DYY1_IAA_YY1_ChIP_Rep4 33004641 22032095

Antibodies

anti-CTCF Abcam ab128873
anti-CTCF Abcam ab128873
anti-YY1 Abcam ab38422
anti-YY1 Abcam ab109237
anti-SMC1A Bethyl laboratories A300-055A
anti-SMC3 Bethyl laboratories A300-060A

Peak calling parameters

ChIP-Seq read alignment: Bowtie (-n 2, -m 1)
ChIP-Seq peak calling: MACS2 (--nomodel --extsize 300)

Data quality

Sample_name Peak_number_FDR5% Peak_number_5-fold
CTCF_degron_auxin_CTCF_IP 26931 18438
CTCF_degron_auxin_Rad21_IP 14989 1129
CTCF_degron_auxin_Smc1a_IP 39093 3210

	CTCF_degron_auxin_Smc3_IP 15393 777<br>CTCF_degron_auxin_YY1_IP 56799 16758<br>CTCF_degron_ut_CTCF_IP 81432 61404<br>CTCF_degron_ut_Rad21_IP 38762 24735<br>CTCF_degron_ut_Smc1a_IP 43472 17216<br>CTCF_degron_ut_Smc3_IP 20194 9213<br>CTCF_degron_ut_YY1_IP 46354 14594<br>Rad21_degron_auxin_CTCF_IP 90823 65960<br>Rad21_degron_auxin_Rad21_IP 11347 2803<br>Rad21_degron_auxin_Smc1a_IP 9511 762<br>Rad21_degron_auxin_Smc3_IP 5464 502<br>Rad21_degron_auxin_YY1_IP 42273 12447<br>Rad21_degron_ut_CTCF_IP 95139 72386<br>Rad21_degron_ut_Rad21_IP 43113 28993<br>Rad21_degron_ut_Smc1a_IP 41091 15957<br>Rad21_degron_ut_Smc3_IP 38509 21803<br>Rad21_degron_ut_YY1_IP 48329 16732<br>WAPL_degron_auxin_CTCF_IP 97574 74792<br>WAPL_degron_auxin_Rad21_IP 49451 33319<br>WAPL_degron_auxin_Smc1a_IP 38650 17317<br>WAPL_degron_auxin_Smc3_IP 45257 26165<br>WAPL_degron_auxin_YY1_IP 40640 12558<br>WAPL_degron_ut_CTCF_IP 98200 75332<br>WAPL_degron_ut_Rad21_IP 43445 29722<br>WAPL_degron_ut_Smc1a_IP 51296 21539<br>WAPL_degron_ut_Smc3_IP 36245 20304<br>WAPL_degron_ut_YY1_IP 40093 13294<br>YY1_degron_auxin_CTCF_IP 101925 75420<br>YY1_degron_auxin_Rad21_IP 45114 29887<br>YY1_degron_auxin_Smc1a_IP 47950 20330<br>YY1_degron_auxin_Smc3_IP 47098 25773<br>YY1_degron_auxin_YY1_IP 14627 3756<br>YY1_degron_ut_CTCF_IP 98775 74506<br>YY1_degron_ut_Rad21_IP 48854 32391<br>YY1_degron_ut_Smc1a_IP 51886 24617<br>YY1_degron_ut_Smc3_IP 46209 28173<br>YY1_degron_ut_YY1_IP 32203 10210
Software	Bowtie2 (v 2.3.5.1), samtools (v 1.9), deepTools (v 3.5.0), MASC2 (v 2.2.6), MAnorm2 (v 1.0),

Flow Cytometry

Plots

Confirm that:

☒ The axis labels state the marker and fluorochrome used (e.g. CD4-FITC).

☒ The axis scales are clearly visible. Include numbers along axes only for bottom left plot of group (a 'group' is an analysis of identical markers).

☒ All plots are contour plots with outliers or pseudocolor plots.

☒ A numerical value for number of cells or percentage (with statistics) is provided.

Methodology

Sample preparation	We measured RFP (mScarletI) intensity for checking YY1 degradation efficiency. YY1-miniIAA7 cells (clone YD39) were treated with 500 uM IAA for time points at 0, 1, 2, 3 hr. Ethanol-treated cells (negative control) were processed with the same procedure.
Instrument	BD Bioscience LSR Fortessa
Software	FlowJo (v10.3), FlowJo LL
Cell population abundance	Cells were detached and dissociated into single cells by trypsin, washed once by culture media, and resuspended into 1 mL of culture media. We typically have >95% of viable cells.
Gating strategy	We gated the main population on the FSC/SSC plot by excluding the apparent populations of cell debris and cell doublets. For RFP (mScarletI) gating, we defined ~99.5% of cells in the untreated cell as RFP-positive cells.

☒ Tick this box to confirm that a figure exemplifying the gating strategy is provided in the Supplementary Information.
