# Supplementary figures and images for: Enhancer–promoter interactions and transcription are largely maintained upon acute loss of CTCF, cohesin, WAPL or YY1
Source: Nat Genet. 2022 Dec 5;54(12):1919–32. doi: 10.1038/s41588-022-01223-8 (PMC9729117; doi:10.1038/s41588-022-01223-8)

# Anti-RAD21 + anti-ACTB

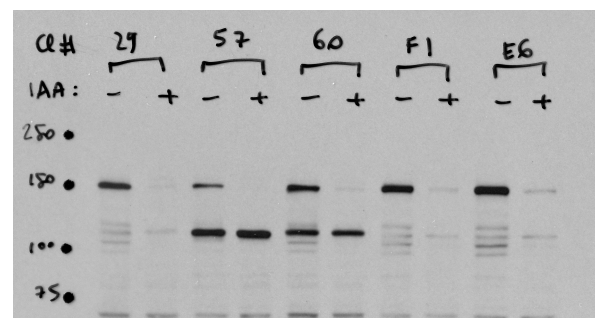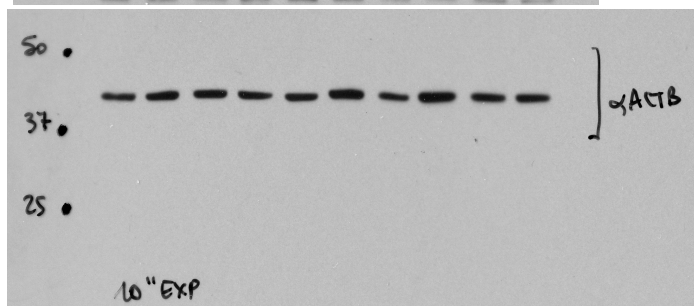

## Anti-CTCF + anti-ACTB

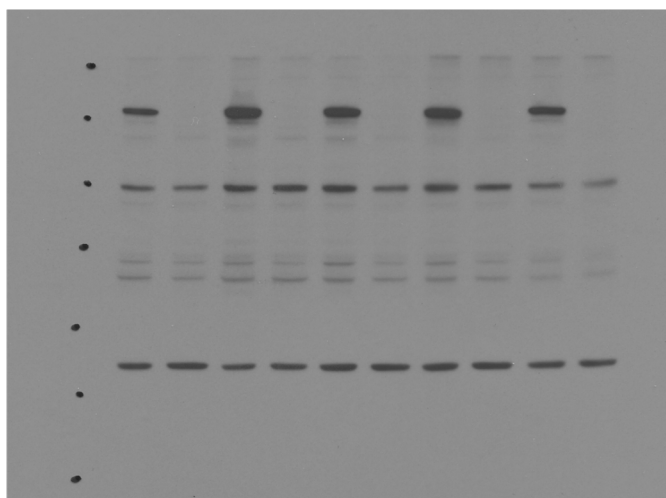

## Anti-HA + anti-ACTB

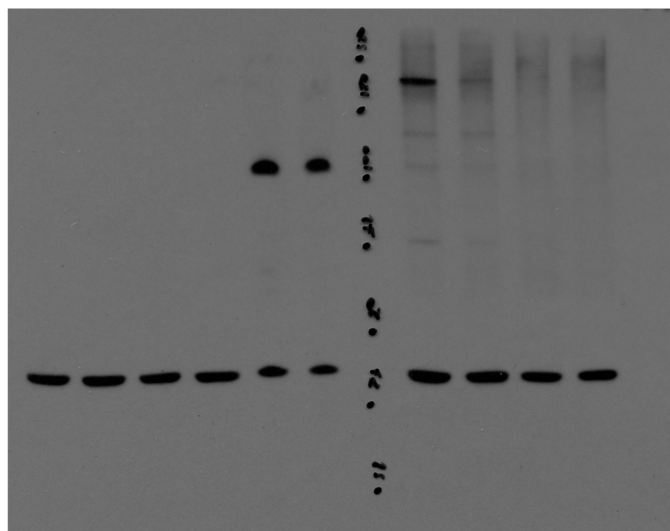

Supplement: Source Data Fig. 3 — Unprocessed western blots. [file 41588_2022_1223_MOESM8_ESM.pdf]

Anti-YY1

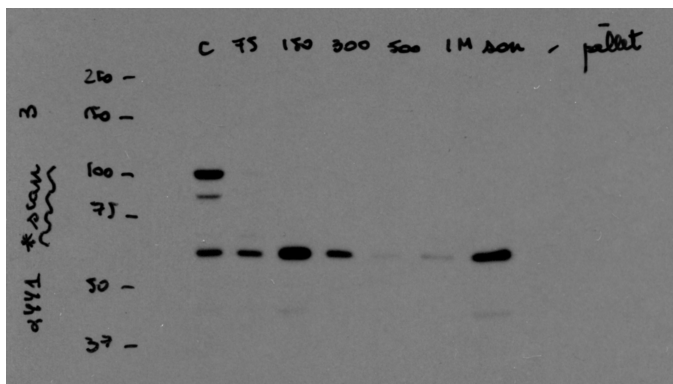

Anti-TBP

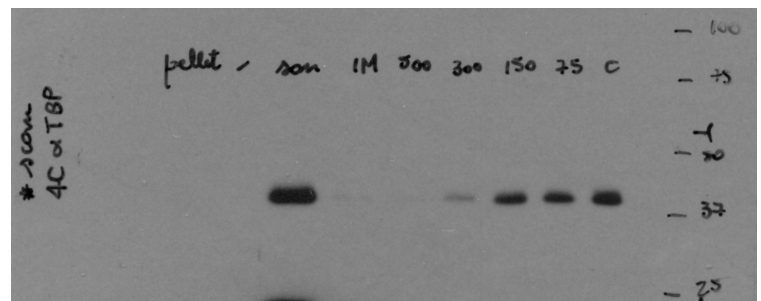

Anti-RAD21

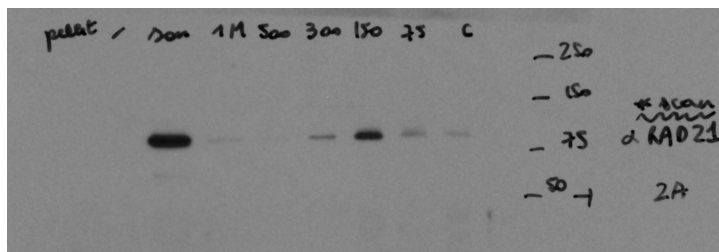

Anti-OCT4

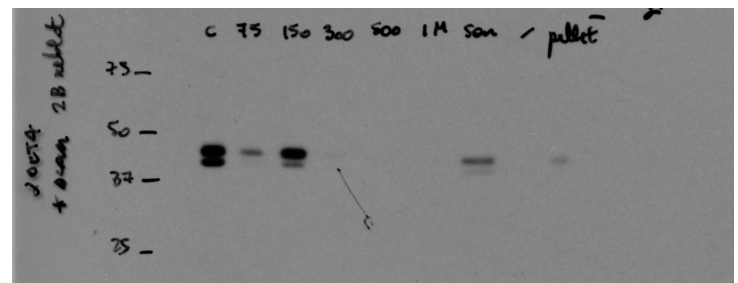

Anti-CTCF

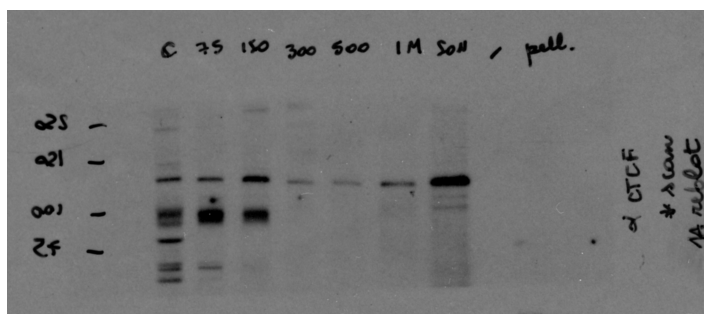

Anti-H2B

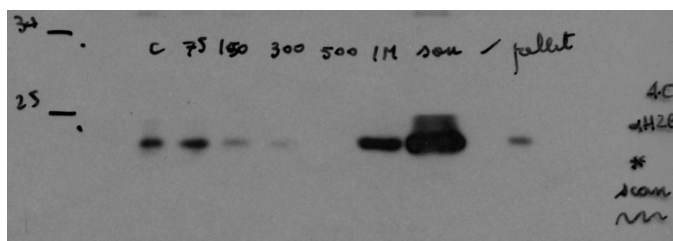

Anti-ACTB

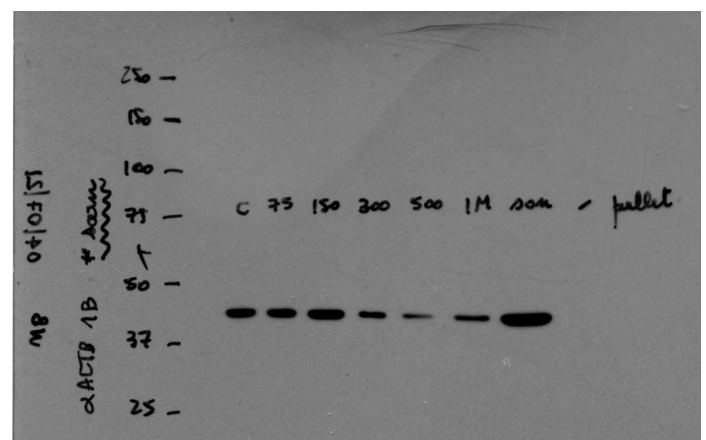

Supplement: Source Data Fig. 6 — Unprocessed western blots. [file 41588_2022_1223_MOESM10_ESM.pdf]

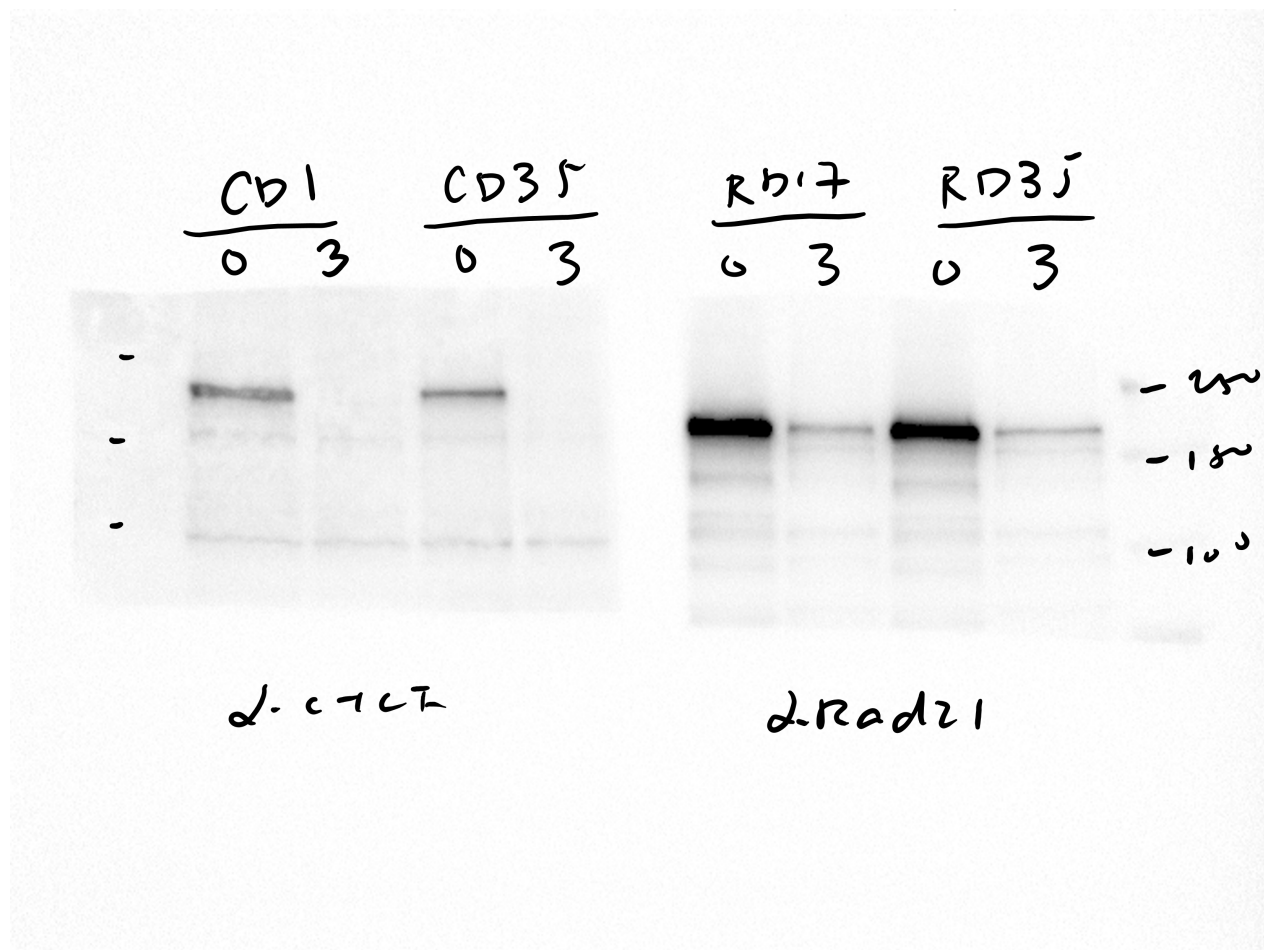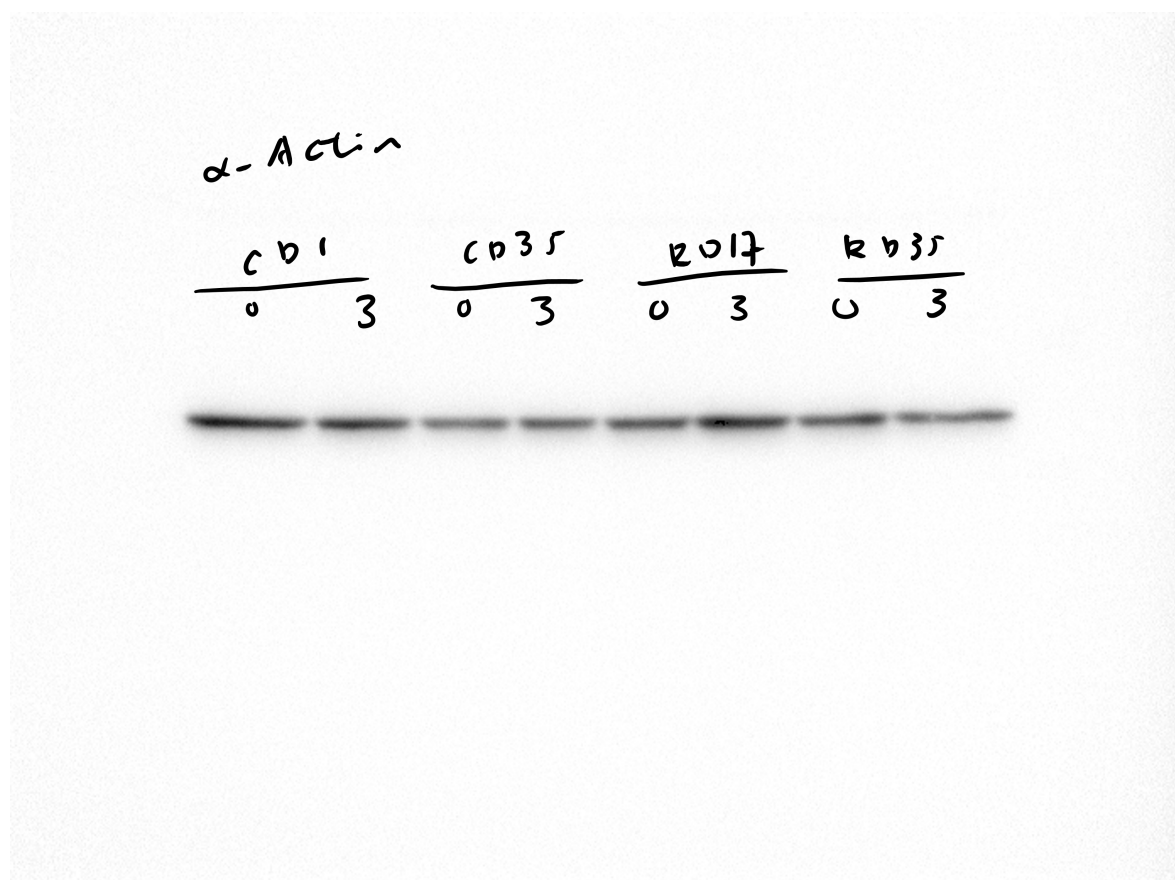

Supplement: Source Data Fig. 7 — Unprocessed western blots. [file 41588_2022_1223_MOESM11_ESM.pdf]

ED\_Fig8c\_Anti-YY1-HaloTag-FLAG

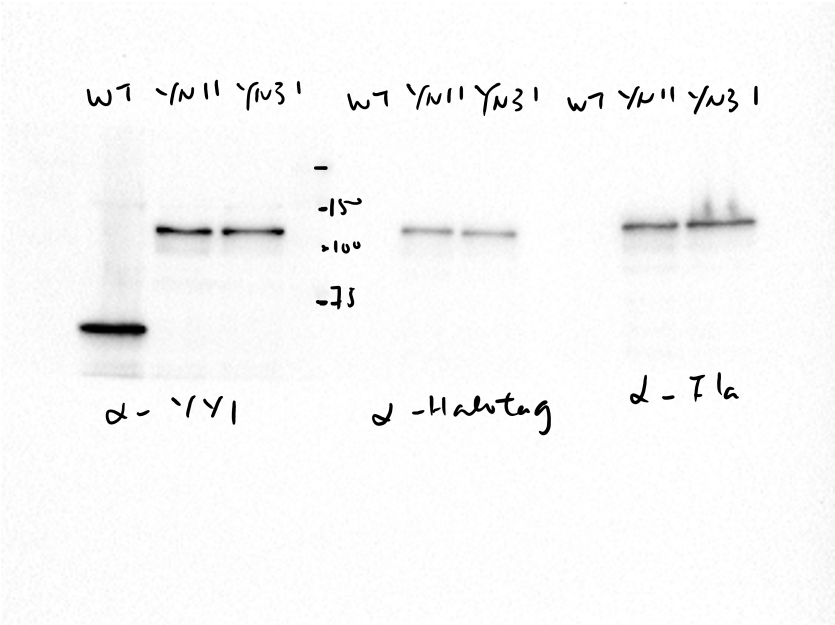

ED\_Fig8c\_Anti-TBP\_right

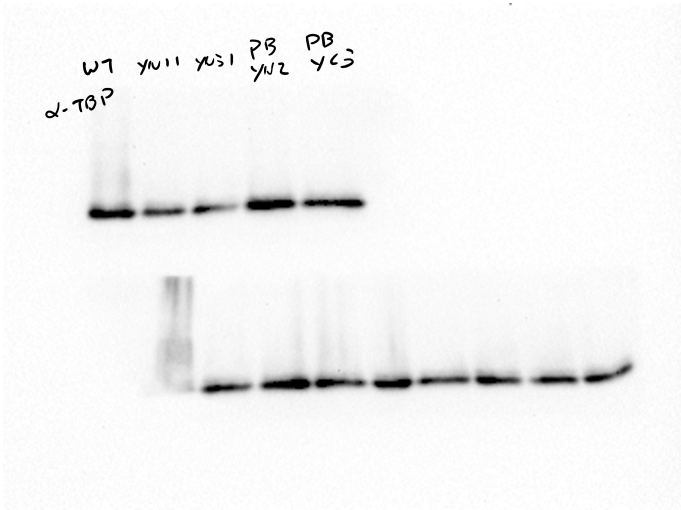

ED\_Fig8c\_Anti-TBP\_left

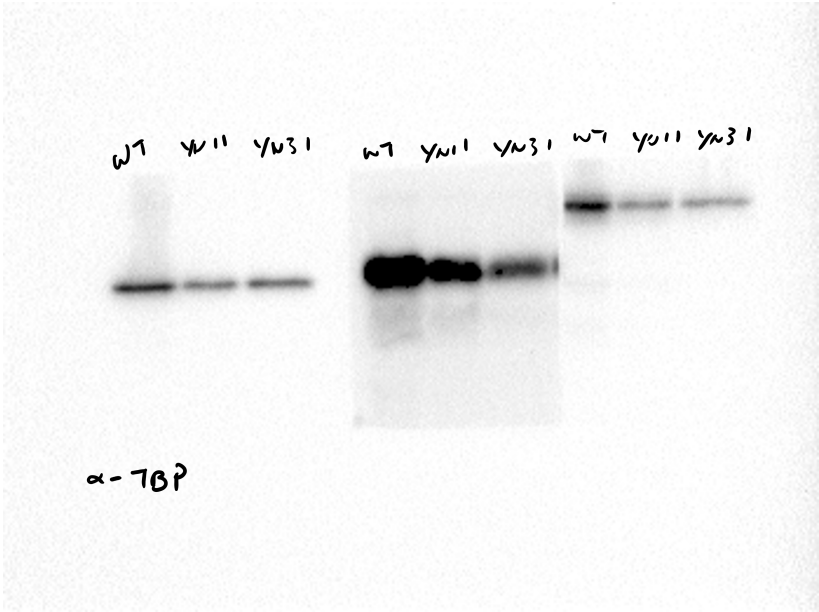

ED\_Fig8c\_Anti-HaloTag

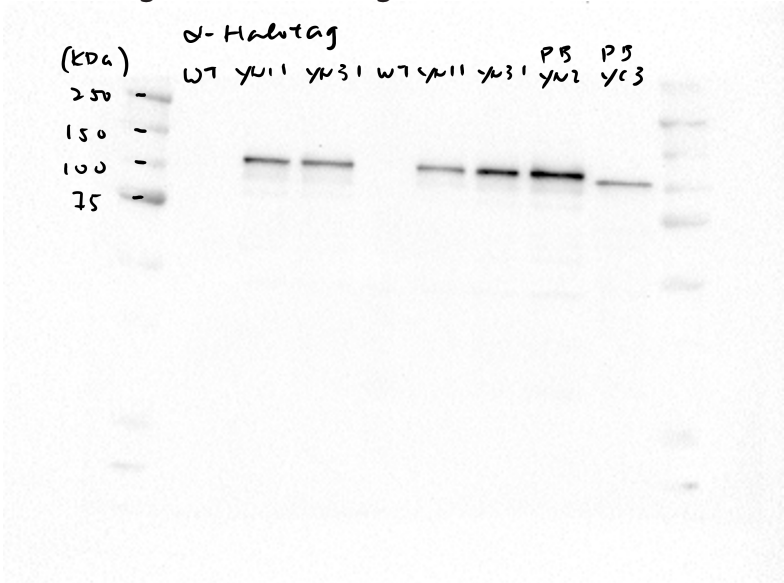

Supplement: Source Data Extended Data Fig. 8 — Unprocessed western blots. [file 41588_2022_1223_MOESM14_ESM.pdf]

ED\_Fig9f\_ΔWAPL\_Anti-YY1 (cut membrane)

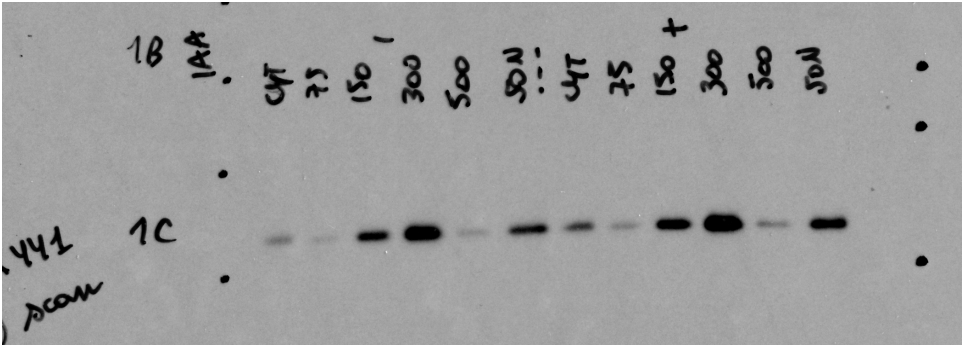

ED\_Fig9f\_ΔCTCF\_ΔRAD21\_Anti-YY1 (cut membrane)

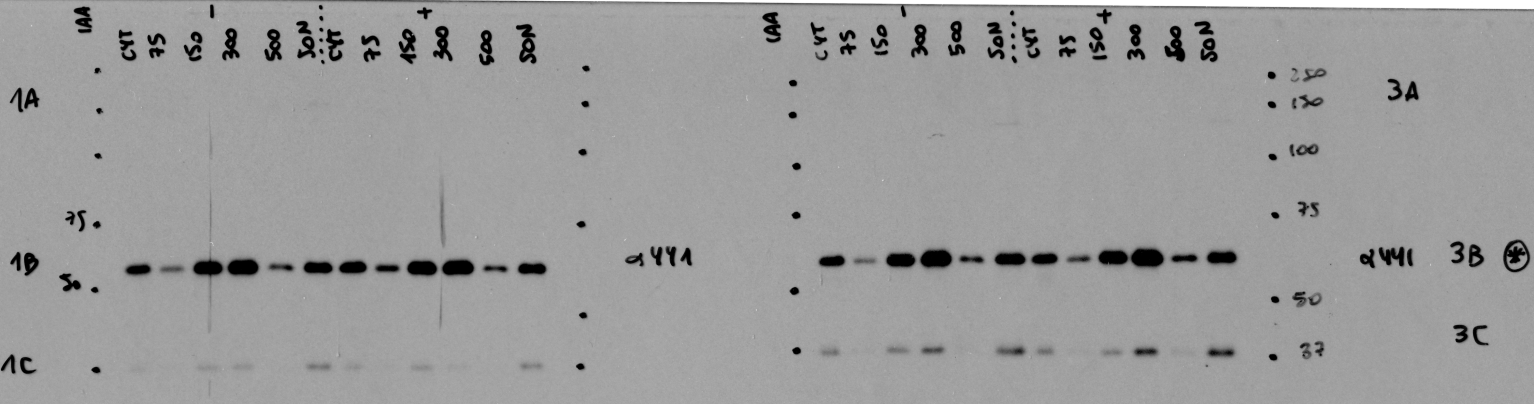

Supplement: Source Data Extended Data Fig. 9 — Unprocessed western blots. [file 41588_2022_1223_MOESM15_ESM.pdf]
