# Supplementary material for: Enhancer–promoter interactions and transcription are largely maintained upon acute loss of CTCF, cohesin, WAPL or YY1
Source: Nat Genet. 2022 Dec 5;54(12):1919–32. doi: 10.1038/s41588-022-01223-8 (PMC9729117; doi:10.1038/s41588-022-01223-8)

ED_Fig2b_Anti-ACTB

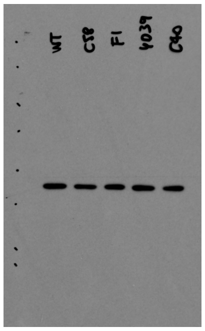

ED_Fig2b_Anti-CTCF (reblotted after ACTB antibody)

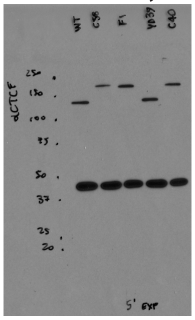

ED_Fig2b_Anti-Halo

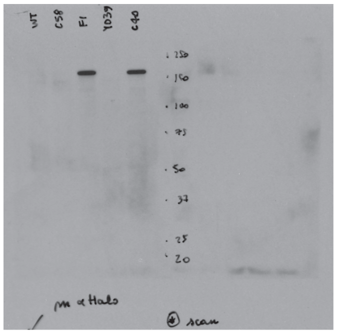

ED_Fig2b_anti-V5-RFP

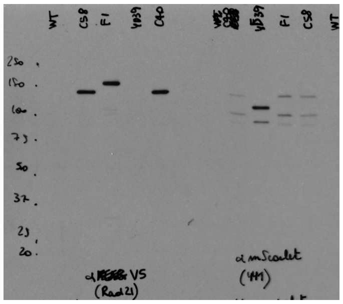

ED_Fig2b_Anti-RAD21-YY1-HA

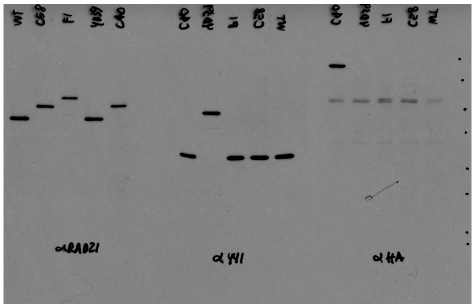

ED_Fig2b_Fig2d_Anti-WAPL

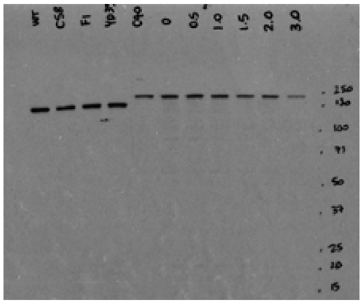

ED_Fig2d_Anti-ACTB (reblotted after WAPL antibody)

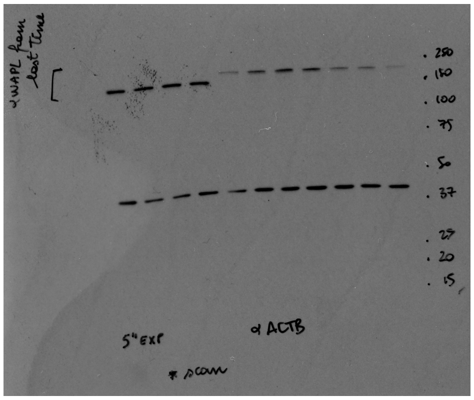

ED_Fig2f_ΔCTCF-Anti-CTCF (cut membrane)

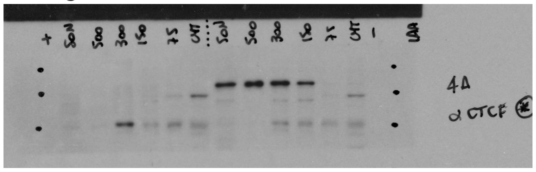

ED_Fig2f_ΔCTCF-Anti-H2B (cut membrane)

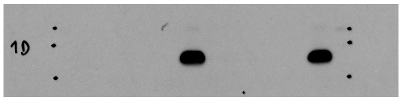

ED_Fig2f_ΔCTCF-ΔRAD21_Anti-RAD21 (cut membrane)

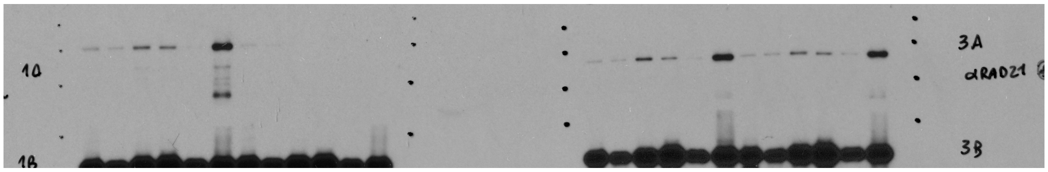

ED_Fig2f_ΔRAD21_Anti-CTCF (cut membrane)

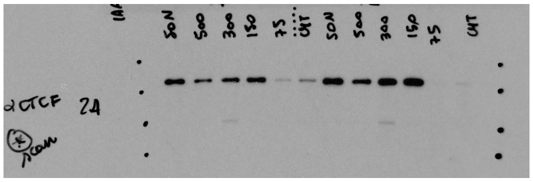

ED_Fig2f_ΔCTCF-ΔRAD21_Anti-WAPL (cut membrane)

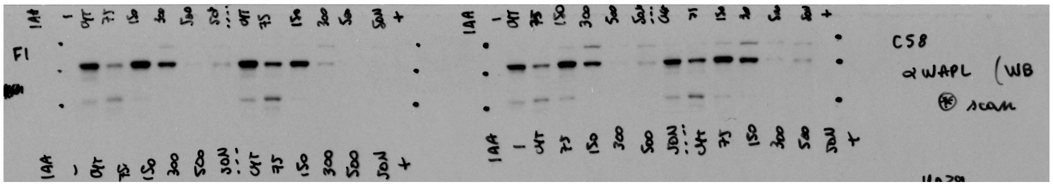

ED_Fig2f_ΔRAD21_Anti-SMC1A (cut membrane)

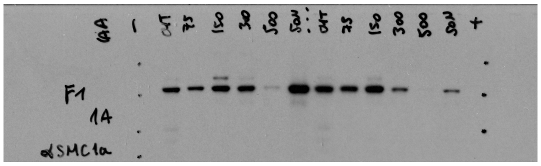

ED_Fig2f_ΔRAD21_Anti-SMC3 (cut membrane)

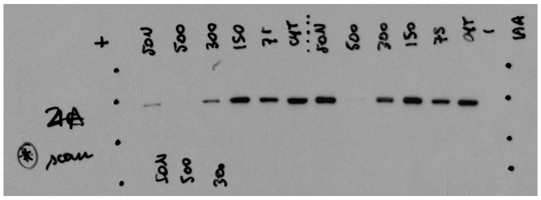

ED_Fig2f_ΔWAPL_Anti-CTCF (cut membrane)

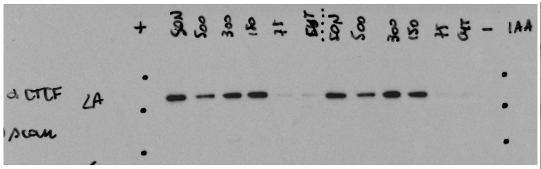

ED_Fig2f_ΔWAPL_Anti-RAD21 (cut membrane)

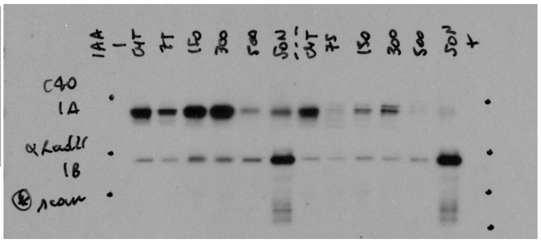

ED_Fig2f_ΔWAPL_Anti-SMC1A (cut membrane)

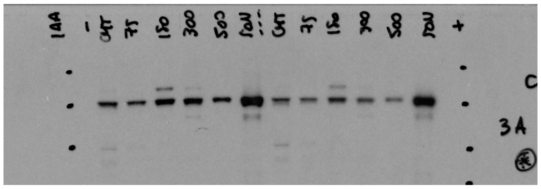

ED_Fig2f_ΔWAPL_Anti-WAPL (cut membrane)

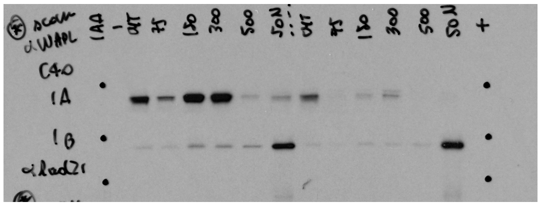

Supplement: Source Data Extended Data Fig. 2 — Unprocessed western blots. [file 41588_2022_1223_MOESM12_ESM.pdf]
