# Supplementary material for: Enhancer–promoter interactions and transcription are largely maintained upon acute loss of CTCF, cohesin, WAPL or YY1
Source: Nat Genet. 2022 Dec 5;54(12):1919–32. doi: 10.1038/s41588-022-01223-8 (PMC9729117; doi:10.1038/s41588-022-01223-8)

ED_Fig7h_ΔYY1_Anti-CTCF (cut membrane)

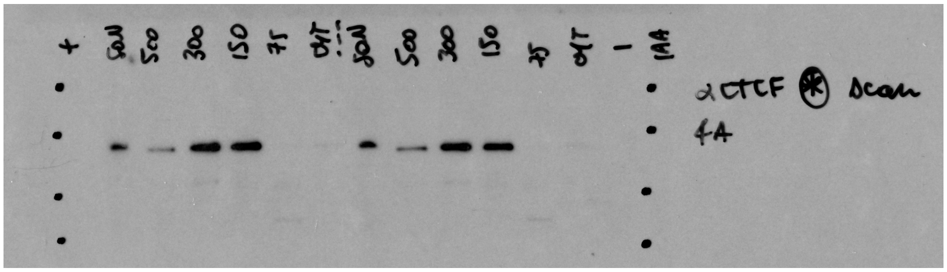

ED_Fig7h_ΔYY1_Anti-RAD21 (cut membrane)

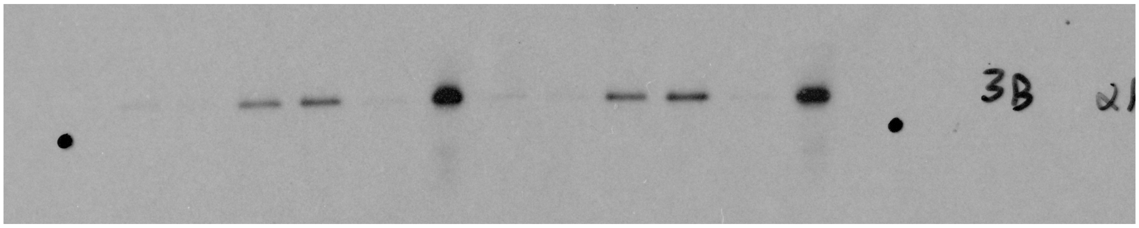

ED_Fig7h_ΔYY1_Anti-WAPL (cut membrane)

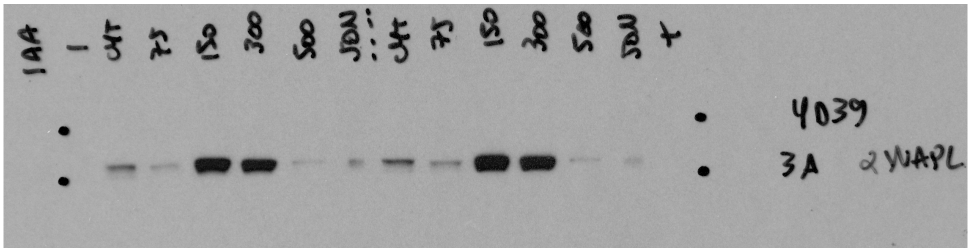

ED_Fig7h_ΔYY1_Anti-YY1 (cut membrane)

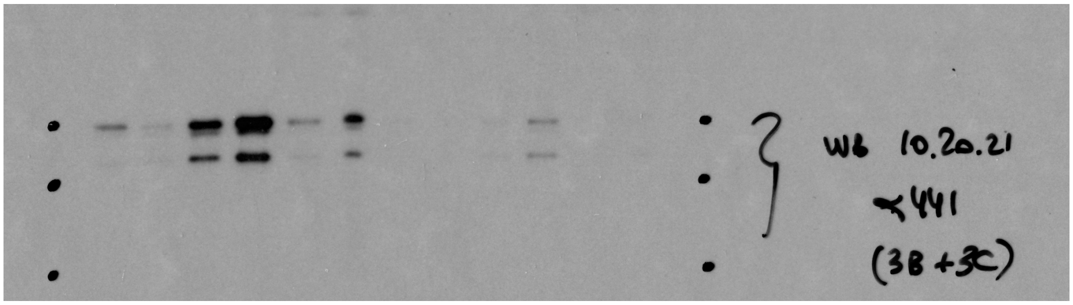

Supplement: Source Data Extended Data Fig. 7 — Unprocessed western blots. [file 41588_2022_1223_MOESM13_ESM.pdf]
